# Square the Circle: Diversity of Viral Pathogens Causing Neuro-Infectious Diseases

**DOI:** 10.3390/v16050787

**Published:** 2024-05-15

**Authors:** Varvara Nurmukanova, Alina Matsvay, Maria Gordukova, German Shipulin

**Affiliations:** 1 Federal State Budgetary Institution “Centre for Strategic Planning and Management of Biomedical Health Risks” of the Federal Medical Biological Agency, 119121 Moscow, Russia; 2 G. Speransky Children’s Hospital No. 9, 123317 Moscow, Russia

**Keywords:** viral neuropathogens, neuroinfections, CNS infections, rare cases, zoonotic infections, vector-borne infections

## Abstract

Neuroinfections rank among the top ten leading causes of child mortality globally, even in high-income countries. The crucial determinants for successful treatment lie in the timing and swiftness of diagnosis. Although viruses constitute the majority of infectious neuropathologies, diagnosing and treating viral neuroinfections remains challenging. Despite technological advancements, the etiology of the disease remains undetermined in over half of cases. The identification of the pathogen becomes more difficult when the infection is caused by atypical pathogens or multiple pathogens simultaneously. Furthermore, the modern surge in global passenger traffic has led to an increase in cases of infections caused by pathogens not endemic to local areas. This review aims to systematize and summarize information on neuroinvasive viral pathogens, encompassing their geographic distribution and transmission routes. Emphasis is placed on rare pathogens and cases involving atypical pathogens, aiming to offer a comprehensive and structured catalog of viral agents with neurovirulence potential.

## 1. Introduction

Neuroinfections is the common name for a polyetiological group of infectious pathologies of the central and peripheral nervous system (CNS and PNS, respectively), characterized by the predominant localization of the infectious agent in certain structural and anatomical areas of the nervous system, and a wide range of clinical manifestations. Meningitis, encephalitis, and myelitis are among the most severe diseases throughout the world [1,2,3,4,5,6].

According to a long-term study of The Global Burden of Disease IHME project [7], despite a downward trend in the global number of deaths observed between 1990 and 2016, the number of reported cases of meningitis worldwide during the same period, on average, increased from 2.50 million to 2.82 million [4,8]. According to data published by a public health organization, Meningitis Research Foundation [9], the total number of estimated cases of meningitis for 2019 worldwide among all age groups was 2.51 million, with a total number of deaths of 236 thousand [10]. However, it is important to note that in this instance, the model for calculating the number of deaths encompasses all causes of meningitis, not solely infectious ones. Furthermore, when compared to mortality estimates for specific infectious causes of death and all causes of death combined, inconsistencies were observed between models in global estimates of mortality from meningitis/encephalitis and neonatal sepsis [3]. The burden is notably heavy in low- and middle-income countries, where prevalent infections like HIV and its related opportunistic infections, Dengue fever, malaria, and various others are widespread, affecting billions of people and often leading to neurological complications [11]. Developing countries experience the greatest burden of meningitis, including 26 countries in the so-called African meningitis belt [3,4] and 4 countries located beyond its borders (India, Pakistan, Afghanistan, and China) [4].

The epidemiological well-being of the child population is of particular concern. Meningitis is one of the ten leading causes of death in children under 14 years of age, even in high-income countries [8]. According to estimates, about 3% of the total number of deaths among children under 5 years of age were caused by meningitis [4]. The diagnosis of aseptic meningitis in children, especially newborns, is difficult; symptoms of the disease in newborns are often subtle and reflect symptoms observed with other infections [4]. Despite differences in the ecological [4,12] and socioeconomic status of the regions of residence [4], age [8], and other factors influencing epidemiology, with different approaches to accounting and building statistical models [3,4,13], there is consensus on the high burden of disease [8], where meningitis ranks second or third in importance among infectious syndromes [3], with the mortality rate reaching 70% without proper treatment [4,8].

The etiology of encephalitis is identified in less than half of cases, both in developed and developing countries, partly due to the lack of standardized approaches to diagnosis [2,6]. Globally, between 1990 and 2019, there was an estimated 12% increase in encephalitis cases, from 1.3 million to 1.4 million, with a total of 89,900 deaths in 2019 [1]. The global incidence of acute encephalitis in adults is estimated to range from 3.5 to 7.5 cases per 100,000 people [14]. The overall incidence among the child population is 16 cases per 100,000 people, but the highest incidence is recorded in children under 1 year of age—8.7 cases [14]; moreover, according to some estimates, approximately one third of cases of childhood encephalitis can be prevented by vaccination [1].

Depending on the ecological and climatic conditions of individual geographical areas, in the etiological structure of encephalitis, there is a predominance of some groups of pathogens over others. For example, tick-borne encephalitis (TBE), transmitted by tick bites, is a serious problem primarily in Europe and then in Asia. However, climate change is gradually leading to an expansion of the habitat of ticks (in general, this also applies to other arthropods), which has allowed tick-borne encephalitis to appear in previously unaffected areas and cause an increase in the number of new cases of encephalitis [1,15].

Inflammatory diseases that are associated with damage to the gray and/or white matter of the spinal cord, such as myelitis of an infectious origin, primarily caused by pathogens of a viral, and then bacterial and other origin [16]. The best-known etiological agent is poliovirus. Poliomyelitis is a severe, highly contagious disease of viral etiology, caused by polio enteroviruses. It is characterized by general paralysis, including respiratory muscles and impaired motor functions, and its mortality rate is 5–10% [17]. Children under 5 years of age are most at risk. However, according to WHO, since 1988, the number of cases of wild poliovirus has decreased by more than 99% [17,18] through immunization programs.

Another variant of myelitis caused by non-polio viruses (in the broad sense, i.e., not only non-polio enteroviruses) is acute flaccid myelitis (AFM) [5,18,19,20]. In case of AFM, damage to the gray matter of the spinal cord is observed, paralysis in the acute phase of the disease, and motor deficit subsequently [5,18,19,20].

To date, there is no systematic tracking of the incidence of the disease, but there are data reflecting the approximate frequency: for example, in the United States, the incidence among children under 15 years of age is estimated at 1.4 cases per 100,000 population per year [18]. In 2014, according to the CDC, there were more than 500 cases [19]. Outbreaks of disease with symptoms consistent with AFM have also been reported in East Asia and Australia, and other regions around the world [5,19].

In most cases, especially without proper treatment, infections caused by neurotropic and neuroinvasive pathogens can rapidly progress and lead to secondary severe conditions, in particular, stroke, epilepsy, and hydrocephalus; subsequently cause neurological deficits and impaired cognitive functions; and be associated with a high risk of death [4,21,22].

Inflammatory processes in the organs of the nervous system can be caused by a wide range of representatives of viruses, bacteria, fungi, protozoa, and helminths [22,23], and in some cases even achlorophyllic algae [24,25], although such cases are much less common [4,22,26]. The early identification of the causative agent is critical for prescribing appropriate etiotropic therapy and informing the patient about subsequent clinical intervention [22]. Often, patients who do not have a diagnosis are treated empirically with broad-spectrum antibiotics [22,27,28]. This, in turn, increases the risk of adverse side effects [29]: the individual reaction of the patient’s body to the drug due to the cumulative effect, even with a normal tolerability of the drug in minimal therapeutic doses [30,31]. And moreover, it generally affects the resistance of pathogens to antimicrobial drugs [27,32].

The key factors determining a positive treatment outcome are the time and speed of diagnosis. Traditional nonspecific diagnostic methods have their own number of advantages and, at the same time, limitations. Therefore, for example, one of the “gold standards” for diagnosing patients with suspected neuroinfection is the analysis of cerebrospinal fluid. Non-specific methods of testing cerebrospinal fluid, including cytochemical analysis and culture on nutrient media, may, in some cases, give a false negative result [33,34,35]. Pathogen-specific methods for detecting pathogens, including a reaction to determine antibodies that indirectly indicate the presence of infection, or the direct detection of nucleic acids using PCR in biomaterial, require the clinician to select the specific pathogen being studied. In some cases, the arsenal of test systems used is limited to widespread pathogens; however, when a rare pathogen is involved in the pathological process, or in the case of combined infections (co- and mixed infections), difficulties arise in determining its taxonomic affiliation [29,36]. Simultaneous analysis with a set of tests is often used, but this significantly increases the diagnostic time to determine the cause of the disease and the final diagnosis [22].

As mentioned earlier, viruses account for the majority of neuropathologies of infectious origin [2,37]; for example, in developed countries, the vast majority of cases of primary encephalitis are caused by viruses [16]. Viral neuroinfections are of great importance due to the ability to occur in a latent form; their ability to activate in case of exacerbation of other infections and/or against the background of chronic diseases, thereby worsening the current condition of the patient; the potential impact on the severity and development of fatal neurodegenerative diseases such as Alzheimer’s disease or Parkinson’s disease [38,39], multiple sclerosis, and amyotrophic lateral sclerosis [40,41]; the development of pathological autoimmune reactions, including those aimed at the central nervous system [42,43]; and long-term physical and cognitive complications [44,45]. Viruses can cause neurological disease directly by infecting and damaging neurons, or indirectly by stimulating an immune response that alters host cell function [46]. Several factors, including neuroinvasiveness, neurotropism, neurovirulence, and immune responses of infected hosts, determine the spectrum and severity of virus-induced neurological signs and symptoms [46,47,48].

Viral lesions of the central nervous system—perinatal, infant and childhood—can lead to the disruption of the development of the nervous system, including the brain, during the formation period [47], or to the development of serious pathologies, including lethal ones, as occurs with congenital infection with lymphocytic choriomeningitis virus [49]. Today, more than 100 types of viruses are known, for which a connection with the development of diseases of the peripheral and central nervous system has been determined [48]. However, the etiology of a significant part of cases remains unclear due to the lack of available diagnostic tests. For some viral pathogens, their role in the development of acute disease and their long-term effect on the nervous system remains unclear. Examples of such controversial diseases are the forgotten lethargic encephalitis (Economo’s disease) [50,51,52], Vilyui encephalomyelitis [53,54], and diseases presumably caused by Betatorqueviruses or *Cyclovirus* genus, sometimes found in biosamples from patients [55,56].

Raising awareness of the etiological structure, particularly concerning rare emerging neurotropic/neuroinvasive viral pathogens, is crucial for enhancing diagnostic capabilities in the realm of infections within this nosology. In this review, we systematized and summarized information about currently known neurotropic viral pathogens and their routes of transmission. Special attention was paid to rare pathogens and cases of infection with atypical pathogens reported in the literature, in order to present the most comprehensive and structured list of viral agents with neuroinvasive potential. This review primarily examines the etiological structure of infectious neuropathologies such as meningitis, encephalitis, and myelitis.

For the convenience of readers, we divided the viral pathogens discussed into the following three groups: (1) viruses whose transmission is predominantly/always carried out with the participation of invertebrate vectors (vector-borne infections); or (2) involves primarily direct contact with a virus-reservoir animal; (3) and common viruses, the transmission of which is predominantly maintained within the human population, from person to person, without the participation of intermediate host vectors. However, it should be noted that the third group of viruses also included representatives of the genera *Mamastrovirus, Betacoronavirus, Alphainfluenzavirus*, *Betainfluenzavirus*, and *Lentivirus*, due to their successful establishment in the population and the possibility of transmission from human to human, despite their active circulation between various vertebrates, and/or confirmed zoonotic origin. Within each group, viral pathogens are considered at the level of species and species variants (subspecies and serotypes), grouped by families and genera, and listed in alphabetical order. At the end of each section, we provided summary tables containing structured information about all pathogens discussed in the section.

## 2. Vector-Borne Viral Neuropathogens

Viruses transmitted by invertebrates are called arboviruses from the words “arthropod-borne”. To date, more than 500 arboviruses have been described, of which about 50 are veterinary-significant, and about 100 are considered potentially pathogenic for humans [57]. The transmission of arboviruses to humans is primarily carried out by ticks and bloodsucking insects of the Diptera order.

### 2.1. Tick-Borne Viral Neuropathogens

Ticks are obligate hematophagous ectoparasites [58]. The two main families of ticks, *Argasidae* (soft ticks) and *Ixodidae* (hard ticks), differ in their ecology and impact on human health [59], in that hard ticks transmit more diseases than soft ticks.

Most viruses that cause neuroinflammation are concentrated within the family *Flaviviridae*. The population living in the habitats of vectors experiences the greatest risk of infection with tick-borne viruses. For example, the Center for Disease Control and Prevention reported [60] more than doubling of tick-borne disease cases in the United States—accounting for more than 75% of all vector-borne disease reports. Outside the United States, tick-borne pathogens are responsible for the majority of vector-borne diseases in temperate Eurasia [59].

***Flaviviridae***. Tick-borne flaviviruses are currently divided into two groups: the group of viruses transmitted by mammalian ticks and the group of viruses transmitted by seabird ticks [61]. Tick-borne encephalitis is one of the leading causes of seasonal morbidity in endemic areas [62]. The clinical course of tick-borne infections can range from asymptomatic to severe or fatal.

**Figure 1:**
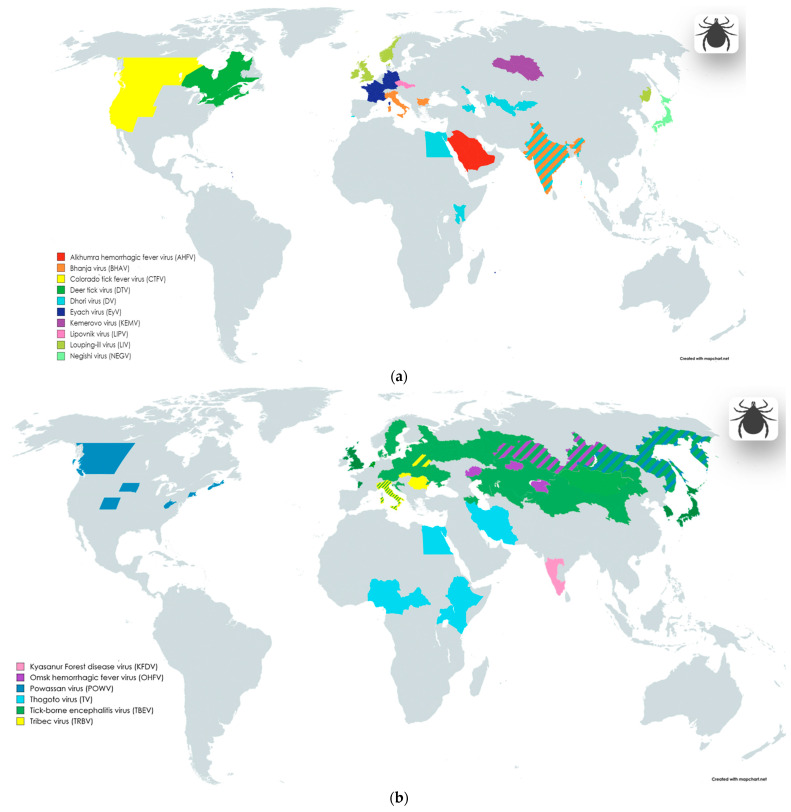
Geographical distribution of tick-borne infections, including areas of endemic and imported cases involving nervous system symptoms. The division into blocks (**a**,**b**) was implemented to enhance the visualization of data on the maps; information about the taxonomic groups located on each block is provided in the legend within the pictures, as well as in the main text alongside the first mention of the corresponding virus. Maps were created with the Map Chart online map-making tool (https://www.mapchart.net/index.html, accessed on 7 May 2024); Graphic objects were created with the BioRender online science illustration making tool (https://www.biorender.com/, accessed on 7 May 2024). The images may contain slight inaccuracies in depicting the boundaries of virus distribution areas due to the limitation of the map scale and the choice of scientific sources utilized to create these illustrations.

The well-known arbovirus **tick-borne encephalitis virus** (**TBEV**) (Table 1, Figure 1b) (previously known as Russian spring and summer encephalitis (RSSE) virus), is widespread throughout different Eurasian geographical zones from Japan to northern Europe, include new endemic areas, for example, South Korea and the Netherlands [63]. Infection with TBEV in humans occurs after the bite of a tick carrying the virus. However, in addition to direct transmission, TBEV can also be transmitted in a food-borne way such as through raw milk [64], if the animal was infected after a bite. The main vectors of TBEV are *Dermacentor reticulatus*, *Ixodes persulcatus*, and *Ixodes ricinus* ticks. TBEV possesses high neurotropicity, and with the development of a severe form of illness, severe neurological syndromes may occur, including meningitis, encephalitis, and acute flaccid paralysis.

**Negishi virus** (**NEGV**) (Table 1, Figure 1a) and **Louping ill virus** (**LIV**) (Table 1, Figure 1a) are similar to each other and closely related variants of the TBEV virus, forming part of the “tick-borne encephalitis (TBE) serocomplex”, as noted in some papers [65,66].

The distribution area of **Negishi virus** (**NEGV**) covers Japan and the adjacent territories of China and the Republic of Korea [61,62,63,64]. The virus was first isolated from the cerebrospinal fluid of a patient in Japan (Tokyo area) in 1948, during an outbreak of Japanese encephalitis [61,62,63,64]. The carriers of NEGV in the territories of Japan, China, and the Republic of Korea are mainly considered ixodid ticks [61,62,63,64]. The exact association and incidence of NEGV encephalitis are difficult to estimate, but awareness is necessary.

The main region of distribution of **Louping-ill virus** (**LIV**) are the areas of the British Isles, particularly Scotland, Cumbria, Wales, Devon, and Ireland [67,68]. However, a closely related LIV variant was also discovered in the Far East of Russia [69]. LIV mainly affects small domestic animals such as sheep and goats; manifestations of the disease in animals include fatal encephalomyelitis and other severe lesions of nervous tissue [66,67,68]. Cases of morbidity in humans have been reported extremely rarely (about 30–45 cases are known, including one with a fatal outcome [67,70]), and people involved in tending livestock and grazing are mainly at risk. In humans, rare manifestations of the disease may include encephalitis and similar conditions [67,68].

**Alkhumra hemorrhagic fever virus** (**AHFV**) (Table 1, Figure 1a) can also be transmitted to humans through soft tick (*Ornithodoros savignyi* or “Sand tampan”) and hard tick (*Hyalomma dromedarii*) bites [71]; however, the possibility of transmission of the virus through mosquito bites and in a food-borne way has also been reported. The Middle East region is recognized as the predominant area for the spread of the virus and its vector [71,72]; AHFV was first isolated from a patient in Mecca (Saudi Arabia), in the mid-1990s [71]. Symptoms of the disease include fever and headache; severe complications are represented by hemorrhagic manifestations and encephalitis, and the mortality rate of the disease is about 20–25% [72,73,74].

**Kyasanur Forest disease virus** (**KFDV**) (Table 1, Figure 1a) was discovered in the 1950s in the state of Karnataka in southern India [73]. In addition to various rodents and birds, susceptible organisms in the wild are monkeys [73]. The infection of humans and animals occurs mainly through the bites of *Haemaphysalis* ticks. However, the detection of the virus among *Ixodes*, argas, *Ornithodoros*, *Hyalomma*, *Dermacentor*, and *Rhipicephalus* ticks has also been reported [73]. Domestic animals such as cows are also bitten [73]. Over time, the number of states in India where outbreaks were recorded among the local population increased [73,75]. In recent decades, an increase in the number of cases has been reported; for example, between 2003 and 2012, a total of more than 3 thousand human cases were reported in Karnataka (of which 28 cases were fatal) [73,75]. Direct person-to-person transmission has not yet been reported. The main manifestation of the disease is fever, sometimes with hemorrhagic and neurological manifestations [73,75]. Approximately 20% of cases have severe symptoms with hemorrhagic and neurological symptoms (including encephalitis) [73,75].

Locally distributed **Omsk hemorrhagic fever virus** (**OHFV**) (Table 1, Figure 1b) is also an important pathogen. The main region of the distribution of OHFV are the Western Siberia parts of Russia [76,77]; however, a group of researchers recently presented a publication that reported the first case of detection of OHFV outside Russia—in the territories of Kazakhstan [78], including areas non-adjacent to Western Siberia. The discovery of a new distribution area for OHFV may be associated with the migration of the main vertebrate hosts—muskrat (*Ondathra zibethicus*) or water vole (*Arvicola amphibius* Syn. *Arvicola terrestris*) [78,79]. The main arthropod hosts of the virus are the ixodid ticks *Dermacentor marginatus* and *Dermacentor reticulatus* [76,79]. In addition, OHFV has been isolated from Aedes and Mansonia mosquito genera, but their role as vectors is minor [76]. The dynamics of incidence since the discovery of OHFV has changed in different periods, with the largest number of cases recorded in the period from 1945 to 1972 [77]. Symptoms of the disease include fever and hemorrhagic manifestations, but a third of patients may develop pneumonia, meningitis, kidney damage, or a combination of manifestations [76].

**Powassan virus** (**POWV**) (Table 1, Figure 1b) is considered an endemic pathogen in North America, causing encephalitis, but several cases of infection with this virus have also been reported in Russia [80,81]. Related to POWV, **deer tick virus** (**DTV**) (Table 1, Figure 1a) is also recognized as a rare but important agent of neuroinfections. The virus was first isolated from the ixodid tick *Ixodes scapularis* in 1997 in North America. The work of Tavakoli et al. [82] describes a case of necrotizing meningoencephalitis with a fatal outcome in an adult patient.

***Orthomyxoviridae***. Two closely related members of this family belonging to the genus *Thogotovirus* are also viruses transmitted by tick bites. **Dhori virus** (**DV**) (Table 1, Figure 1a) and **Thogoto virus** (**TV**) (Table 1, Figure 1b) remain relatively poorly studied, but have been reported to cause a range of clinical manifestations, from self-limited febrile state to meningoencephalitis and encephalitis [83,84].

**Dhori virus** (**DV**) was originally isolated from *Hyalomma dromedarii* camel ticks in Dhori state, India [84]. In addition to India, the virus has also been detected in carriers in the Astrakhan region of Russia, in southern Portugal, Central Asia, Kenya, etc. Dhori virus was also found in mosquitoes; a number of authors do not exclude the possibility of transmission of this pathogen from human to human [84].

**Thogoto virus** (**TV**) was first isolated from *Boophilus decoloratus* and *Rhipicephalus* spp. ticks, collected from cattle in the Thogoto Forest in Nairobi, Kenya, in 1960. In Europe, this virus was discovered a few years later in Sicily in 1969. Reports of human cases included bilateral optic neuritis and a case of fatal meningoencephalitis associated with hepatitis [84].

***Phenuiviridae*** are a large family of RNA-containing arboviruses, pathogenic for humans and animals, currently including 19 recognized genera and more than 100 species [85]. Phenuiviruses have a wide geographic distribution, and the range of competent vectors includes ticks and dipterous insects [85].

A representative of the family, **Bhanja virus** (**BHAV**) [79,84,86,87] (Table 1, Figure 1a) (*Bandavirus*) was first isolated from *Haemaphysalis intermedia* ticks in Banjanagar, India, in 1954 [84]. The virus is now known to be transmitted by ixodid ticks of several genera such as *Amblyomma*, *Boophilus*, *Dermacentor*, *Haemaphysalis*, and *Rhipicephalus*. The prevalence of BHAV ticks is not limited to India. In competent hosts, the virus is found in areas with warm climates and steppe vegetation, in Central Asia, Europe (in regions of Italy and Bulgaria), Africa, and several regions of Southeast Asia [84]. In humans, BHAV causes a febrile illness, sometimes with meningoencephalitis or encephalitis, photophobia, vomiting, and paresis.

***Sedoreoviridae***. The genus *Orbivirus* of the family contains more than 100 subspecies, classified into 14 serogroups, infecting a wide range of arthropod and vertebrate hosts [88,89]. Most members of this genus infect vertebrates but not humans, although transmission to humans is thought to be possible. Recognized vectors involved in the transmission of these viruses include a wide range of invertebrate intermediate hosts, such as mosquitoes, midges, and ticks [88,89].

For humans, three species of orbiviruses pose a danger as etiological agents of neuroinflammation, namely, **Kemerovo virus** (**KEMV**) (Table 1, Figure 1a), **Lipovnik virus** (**LIPV**) (Table 1, Figure 1a), and **Tribec virus** (**TRBV**) (Table 1, Figure 1b), common in Russia and Eastern Europe [88,89].

Ticks of the genus *Ixodes* in Russia (Western Siberia is an endemic region) transmit **Kemerovo virus** (**KEMV**). This virus has also been detected in migrating birds (*Phoenicurus phoenicurus*) in Egypt, and in *Hyalomma anatolicum* ticks in Uzbekistan, which indicates a more complex and widespread distribution of the pathogen [88,89,90]. In humans, KEMV infection is associated with the development of aseptic meningitis, meningoencephalitis, and encephalitis [88,89,90].

**Lipovnik virus** (**LIPV**) is also common in *Ixodes ricinus* ticks. Presumably, competent amplification hosts are various wild forest rodents living in the territories of Slovakia and the Czech Republic. In case of an unfavorable outcome, after being bitten by an infected tick, a person also develops aseptic meningitis, meningoencephalitis, or encephalitis [58,88,89,90].

**Tribec virus** (**TRBV**) is transmitted by ixodid ticks in the territories of Slovakia, Moldova, Romania, Belarus, and Italy. Amplification hosts are small wild rodents: the virus has so far been found in voles and hares. The virus has also been detected in migrating birds and domestic goats. Infection occurs after a tick bite, and can subsequently lead to meningitis, meningoencephalitis, or encephalitis, as is the case with the KEMV and LIPV viruses [58,88,89,90].

***Spinareoviridae***. **Colorado tick fever virus** (**CTFV**) (Table 1, Figure 1a) and **Eyach virus** (**EyV**) (Table 1, Figure 1a), belonging to the genus *Coltivirus*, have been described as agents that cause febrile illnesses and neuroinfections.

The range of vectors of the **Colorado tick fever virus** include forest hard ticks of the *Dermacentor* genus, *Haemaphysalis leporispalustris* species, and *Ixodes* genus, and the soft tick *Otobius lagophilus* [52,84,91,92,93]. The main regions of distribution of these viruses are North America for CTFV [84] and Europe for EYAV [94,95]. In addition to direct transmission through a tick bite, CTFV may also be transmitted from person to person, through blood transfusion, or congenitally through maternal infection [94]. Severe manifestations of CTFV infection may include aseptic meningitis, encephalitis, and meningoencephalitis [52,84,96].

**Eyach virus** was isolated from *Ixodes ricinus* and *Ixodes ventalloi*, and the clinical manifestations of the infection include meningoencephalitis [58,59,84,94,95].

**Table 1:** List of tick-borne viral pathogens of neuroinflammation.

Family	Genus	Species/ Acronym(s)	Common Names or Subspecies/ Acronym(s)	Genome	Host-Source, Vector, Transmission	Geographic Distribution	NS Pathology	Reference
		(Sub)Species Complex/ Acronym(s)						
*Flaviviridae* *Flaviviridae*	* Orthoflavivirus *	* Orthoflavivirus * *encephalitidis*	Tick-borne encephalitis virus/ TBEV	ssRNA(+)	Hs: Sylvatic birds, rodents, domestic ruminants V: Hard ticks (*Dermacentor reticulatus*, *Ixodes persulcatus*, *Ixodes ricinus*) T: With tick bites—primarily; Food-borne way (raw milk and dairy products); SOT—rare cases.	Highly endemic regions: China (Inner Mongolia, Northwestern parts of China), Russia, Belarus, Ukraine, Croatia, Poland, Baltic countries, Czech Republic, Southern Germany, Austria, Sweden New endemic areas: France (Bordeaux region), Italy, Japan, Netherlands, England, South Korea, Mongolia, Denmark, Kazakhstan, Kyrgyzstan, Armenia, Azerbaijan, Uzbekistan	Meningitis, encephalitis, Meningoencephalitis, encephalitis, meningitis, poliomyelitis like flaccid paralysis, polyradiculoneuritis	[46,62,63,79,97,98,99,100,101,102]
			Negishi virus ^1^/ NEGV	ssRNA(+)	Hs: Small mammals (large Japanese field mouse—*Apodemus speciosus*; small Japanese field mouse—*Apodemus argenteus*; grey red-backed vole—*Myodes rufocanus*; brown rat—*Rattus norvegicus*)—presumably V: Hard ticks (*Ixodes ovatus*—presumably) T: With tick bites	Japan	Encephalitis	[52,103,104]
		* Orthoflavivirus loupingi *	Louping-ill virus/ LIV	ssRNA(+)	Hs: Sheep, goats, domestic sheep dog, yellow-necked mouse (*Apodemus sylvaticus*), common shrew (*Sorex araneus*), mountain hare (*Lepus timidus*), red grouse (*Lagopus lagopus scoticus*) V: Hard ticks (*Ixodes ricinus*) T: With tick bites—primarily; Food-borne way (raw milk); Contact with contaminated animal blood; Laboratory-acquired infection.	England, Scotland, Ireland, Norway, Denmark (Bornholm), Russian Federation (Primorsky Krai)	Encephalitis	[52,67,69]
		* Orthoflavivirus * *kyasanurense*	Alkhumra hemorrhagic fever virus/ AHFV	ssRNA(+)	Hs: Camels, sheep V: Hard ticks (*Hyalomma dromedarii)*, soft ticks (*Ornithodoros savignyi*) T: With tick bites—primarily; Food-borne way (raw milk); Contact with contaminated animal blood.	Highly endemic regions: Saudi Arabia, Egypt	Encephalitis	[72,73,74]
			Kyasanur Forest disease virus/ KFDV	ssRNA(+)	Hs: Black-faced langur (genus *Semnopithecus*), red-faced bonnet macaque (*Macaca radiate*); forest rats, shrews, white-bellied rat (*Niviventer niviventer*), squirrels, bats (*Rhinolophus rouxi*), ground-dwelling birds, Indian crested porcupines (*Hystrix indic*) V: Hard ticks (*Haemophysalis spinigera*) T: With tick bites	India (Goa, Karnataka, Kerela, Maharashtra, Tamilnadu states)	Encephalitis, aseptic meningitis-like picture	[73,75]
		* Orthoflavivirus * *omskense*	Omsk hemorrhagic fever virus/ OHFV	ssRNA(+)	Hs: Muskrats (*Ondatra zibethicus*), water vole (*Arvicola terrestris*); other local species of rodents V: Hard ticks (*Dermacentor reticulatus*, *Dermacentor marginatus*—primarily; *Ixodes persulcatus*, *Ixodes apronophorus*—rarely) T: With tick bites—primarily; Contact with blood and raw muskrat leather-material—rarely.	Highly endemic regions: Russia (Kurgan, Omsk, Tyumen, Novosibirsk regions); Kazakhstan (Almaty region (human CSF sample), Akmola region (ticks), West Kazakhstan (rodents))	Encephalitic symptoms (continuous headache and meningism)	[76,78,79]
		* Orthoflavivirus * *powassanense*	Powassan virus/ POWV	ssRNA(+)	Hs: Woodchuck (*Mormota monax*)—main reservoir; skunk (*Mephitis mephitis*); sylvatic wild rodents; carnivores V: Hard ticks *Dermacentor andersoni*—Colorado; *Haemaphysalis neumanni*—Primorsky Krai, Russia T: With tick bites	Highly endemic regions: Russia (Far East); US (Colorado, Connecticut, Massachusetts, South Dakota, West Virginia); Canada (Alberta, British Columbia, Nova Scotia)	Meningitis, encephalitis, encephalomeningitis	[79,80,104,105,106,107]
			Deer tick virus/ DTV	ssRNA(+)	Hs: White-footed mouse (*Peromyscus leucopus*)—main reservoir; sylvatic wild rodents; carnivores V: Hard ticks (*Dermacentor andersoni*, *Ixodes scapularis*) T: With tick bites	North US (Hudson Valley, Nantucket Island, Prudence Island); Canada	Encephalitis, meningopolio-encephalitis, meningopoliomyelitis	[82,106,107]
* Orthomyxoviridae *	* Thogotovirus *	* Thogotovirus * *dhoriense*	Dhori virus/ DV	ssRNA(−)	Hs: Banded mongooses (*Mungos mungo*); wild and domestic rodents; domestic ruminants V: Hard ticks (*Amblyomma gemma*, *Hyalomma marginatum*, *Hyalomma dromedarii*); may be transmitted by mosquitoes (*Anopheles hyrcanus*, *Aedes caspius*, *Culex hortensis*) T: With tick bites	Focally endemic worldwide spread in natural boskematic foci; Southern Portugal, Egypt, Astrakhan (Volga delta), Kenya (eastern and northeastern provinces), India, Armenia, Azerbaijan, Kirghizia, Uzbekistan	Meningoencephalitis, encephalitis-like reaction, encephalitis	[83,84]
		* Thogotovirus thogotoense *	Thogoto virus/ TV	ssRNA(−)	Hs: Cattle, camels V: Hard ticks (*Amblyomma variegatum*, *Hyalomma anatolicum*, *Hyalomma eruncatum*, *Rhipicephalus appendiculatus*, *Rhipicephalus evertsi*, *Rhipicephalus sanguineus*) T: With tick bites	Nigeria, Kenya, Uganda, Ethiopia, Cameroon, Central Africa, Egypt, Iran	Bilateral optic neuritis, fatal meningoencephalitis	[84]
* Phenuiviridae *	* Bandavirus *	* Bandavirus * *bhanjanagarense*	Bhanja virus/ BHAV	ssRNA(+/−)	Hs: Cattle, sheep, goats V: Hard ticks (*Amblyomma variegatum*, *Boophilus annulatus*, *Boophilus decoloratus*, *Boophilus geigyi*, *Dermacentor marginatus*, *Haemaphysalis salctata*, *Rhipicephalus bursa*, *Rhipicephalus appendiculatus*, etc.) T: With tick bites	Focally endemic worldwide spread in natural boskematic foci; Europe (Italy, Bulgaria); India	Meningoencephalitis, encephalitis, paresis	[79,84,86,87]
* Sedoreoviridae *	* Orbivirus *	*Great Island virus/* GIV	Kemerovo virus ^1^/ KEMV	dsRNA	Hs: Migrating bird (redstarts—*Phoenicurus phoenicurus*, in Egypt) V: Hard ticks (*Ixodes persulcatus*—Russia; *Hyalomma anatolicum*—Uzbekistan) T: With tick bites	Highly endemic region: Western Siberia (Kemerovo region) Egypt (from migratory birds)	Aseptic meningitis, meningoencephalitis, encephalitis	[88,89,90]
			Lipovnik virus ^1^/ LIPV	dsRNA	Hs: Sylvatic rodents? V: Hard ticks (*Ixodes ricinus*) T: With tick bites	Slovakia, Czech Republic	Aseptic meningitis, meningoencephalitis, encephalitis	[58,88,89,90]
			Tribec virus ^1^/ TRBV	dsRNA	Hs: Rodents (bank vole—*Clethrionomys glareolus*; pine vole—*Microtus pinetorum*; hare—*Lepus europeus*); goats; birds (European starling—*Sturnus vulgaris*; common chaffinch—*Fringilla coelebs*) V: Hard ticks (*Ixodes ricinus* – Czechoslovakia, Moldova; *Haemaphisalis punctate* –Romania) T: With tick bites	Slovakia, Moldova, Romania, Italy, Belarus	Aseptic meningitis, meningoencephalitis, encephalitis	[58,88,89,90]
* Spinareoviridae *	* Coltivirus *	* Colorado tick * *fever coltivirus*	Colorado tick fever virus/ CTFV	dsRNA	Hs: Golden-mantled ground squirrels (*Callospermophilus lateralis*); chipmunks (*Tamias* spp.) V: Wood hard tick (*Dermacentor andersoni*); also hard ticks (*Dermacentor albopictus*, *Dermacentor arumapertus*, *Dermacentor occidentalis*, *Haemaphysalis leporispalustris, Ixodes sculptus*, *Ixodes spinipalpis*); also soft ticks (*Otobius lagophilus*) T: With tick bites; with blood transfusion (from infected humans)	Highly endemic region: Western parts of North US; Canada (Alberta, British Columbia)	Aseptic meningitis, encephalitis, meningoencephalitis	[52,84,96]
		* Eyach coltivirus *	Eyach virus/ EyV	dsRNA	Hs: European rabbit (*Oryctolagus cunniculus*) V: Hard ticks (*Ixodes ricinus*, *Ixodes ventalloi*) T: With tick bites	Germany, France	Meningoencephalitis	[58,59,84,94,95]

Taxonomic and trivial names are given according to the reports of the International Committee on Taxonomy of Viruses (ICTV) https://ictv.global/msl and https://ictv.global/vmr (versions from 2022/2023); ^1^ Commonly accepted alternative names not given in the reports of the International Committee on Taxonomy of Viruses (ICTV) (see above); **SOT**—Solid organ transplantation.

### 2.2. Mosquito- and Midge-Borne Viral Neuropathogens

Diptera, such as mosquitoes and midges, are even more effective agents of arbovirus transmission. The dynamics of distribution are influenced by many factors, but given the rapid climate change and anthropogenic influences, there is a risk of expansion of the original habitats of vectors, including dipterans, for example, through the inclusion of new intermediate hosts such as rodents [108] or birds [79].

**Figure 2:**
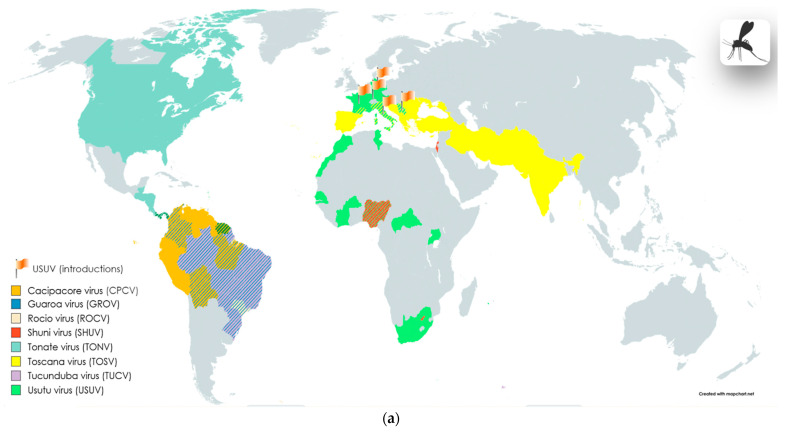

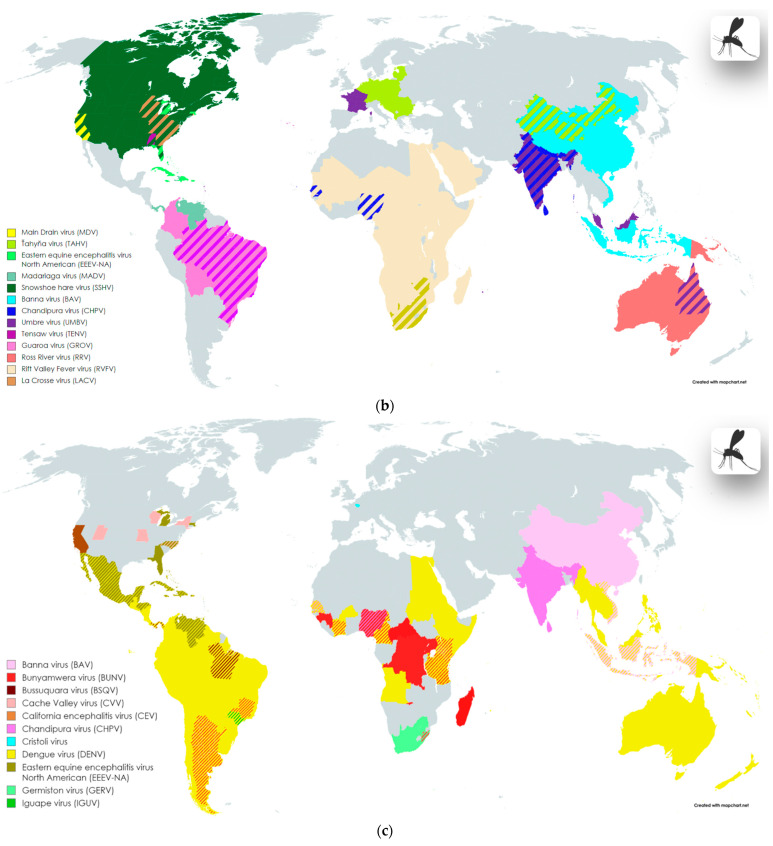

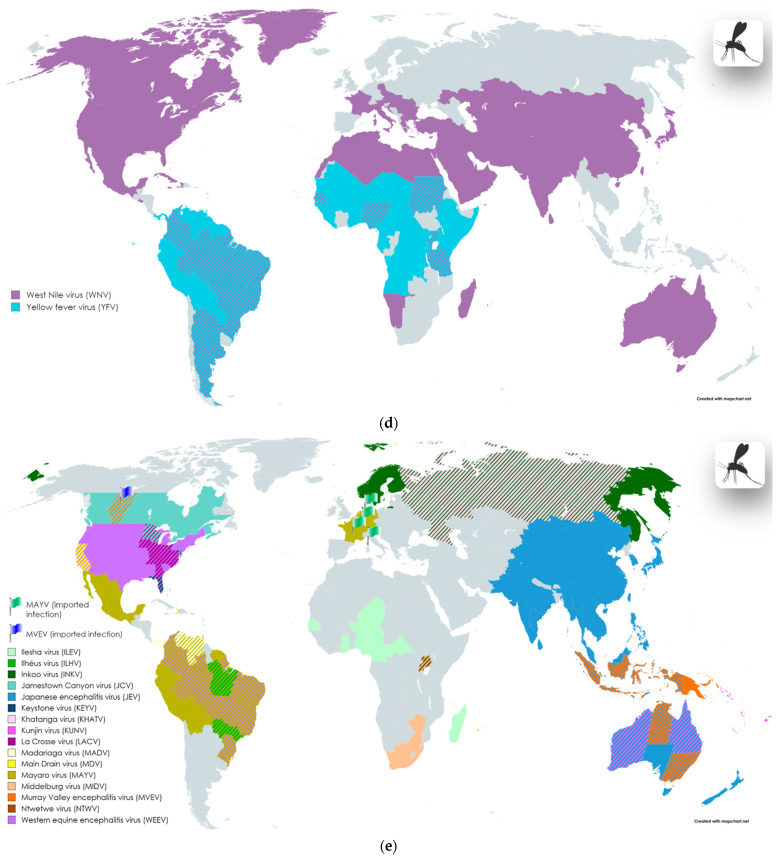

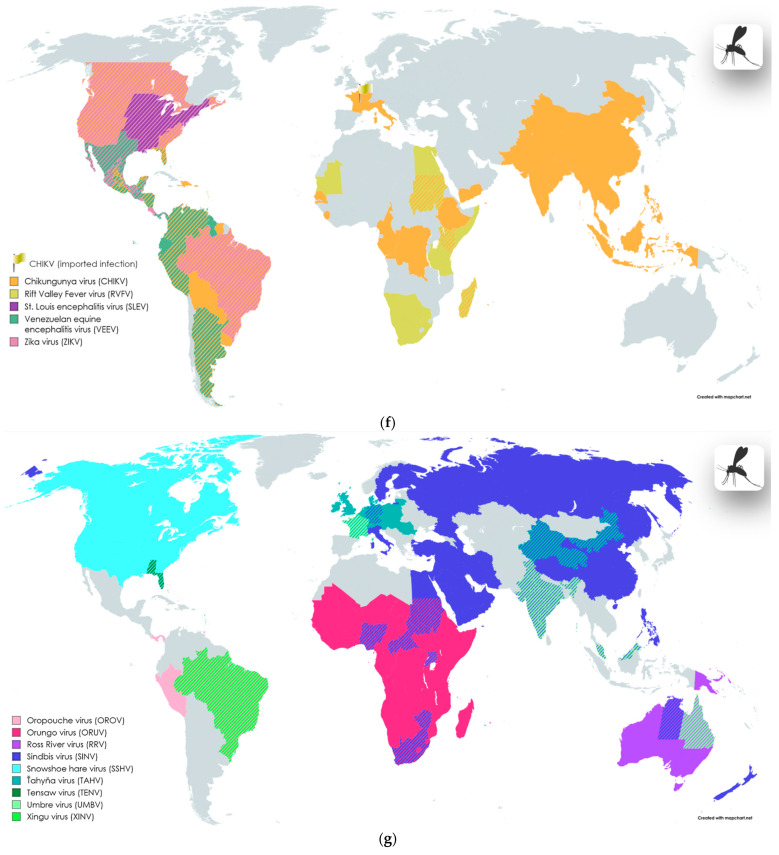
Geographic distribution of mosquito- and midge-borne infections, including areas of endemic and imported cases involving nervous system symptoms. The division into blocks (**a**–**g**) was implemented to enhance the visualization of data on the maps; information about the taxonomic groups located on each block is provided in the legend within the pictures, as well as in the main text alongside the first mention of the corresponding virus; imported cases are marked with flags. Maps were created with the Map Chart online map-making tool (https://www.mapchart.net/index.html, accessed on 7 May 2024); graphic objects were created with the BioRender online science illustration making tool (https://www.biorender.com/, accessed on 7 May 2024). The images may contain slight inaccuracies in depicting the boundaries of virus distribution areas due to the limitation of the map scale and the choice of scientific sources utilized to create these illustrations.

***Flaviviridae***. **Bussuquara virus** (**BSQV**) (Table 2, Figure 2c), **Iguape virus** (**IGUV**) (Table 2, Figure 2c), and **Cacipacoré virus** (**CPCV**) (Table 2, Figure 2a) are mainly transmitted to humans through the bites of infected *Aedes* mosquitoes in endemic regions of Brazil. Like other viruses classified in the genus, CPCV, BSQV, and IGUV have important medical significance as pathogens capable of causing, in addition to fever, severe manifestations including encephalitis [109,110]. The causative agent of Dengue fever, **Dengue virus** (**DENV**) (Table 2, Figure 2c) is transmitted through the bites of mosquitoes of the genus *Aedes* spp. The greatest burden of disease from DENV is experienced by populations living in tropical and subtropical regions (the number of human cases of infection is estimated to range from 100 to 400 million) [111]. DENV usually occurs in a mild or asymptomatic form, but in some cases, the disease can progress to hemorrhagic fever and cause encephalopathy, polyneuritis, cerebellitis, etc.

**Yellow fever virus** (**YFV**) (Table 2, Figure 2d) circulates in a cycle between non-human primates and mosquitoes of the genera *Aedes*, *Haemagogus*, and *Sabethes* [112,113,114,115,116,117,118]. The transmission of the virus to humans and non-human primates occurs after the bite of an infected insect, but also, in some regions of Africa, cases of transmission through the bites of the *Amblyomma variegatum* tick have been reported [112,113,114,115,116,117,118]. The main regions where the virus spreads are Africa and South America. In some cases, Yellow fever virus infection may be characterized by systemic damage, including the liver, kidneys, and nerve tissue. Nervous system complications of yellow fever include encephalitis (including YFV vaccine-associated encephalitis), ADEM, and meningitis [112,113,114,115,116,117,118].

**Ilheus virus** (**ILHV**) and **Rocio virus** (**ROCV**) are important etiological agents of encephalitis in South America. **Ilheus virus** (**ILHV**) Table 2, Figure 2e) has been reported to cause severe febrile illness, and cases of encephalitis have been reported in Central and South America and Trinidad [109]. **Rocio virus** (**ROCV**) (Table 2, Figure 2a) is associated with a large outbreak of epidemic encephalitis in the 1970s, recorded in southeast Brazil [109]. ROCV is transmitted by mosquitoes of the genus *Ochleratus* from the reservoir host—*Zonotrichia capensis* sparrows; however, the complete epidemiological cycle of this virus is not precisely defined [119].

**Japanese encephalitis virus** (**JEV**) (Table 2, Figure 2e) is transmitted through the bite of *Culex spp.* The endemic area for the spread of the virus is the Asia–Pacific region. The virus circulation cycle is associated with wild waterfowl and domestic animals [120]. According to the WHO, the annual number of cases of the disease reaches 70 thousand, and the mortality rate is estimated at 20–30% [120].

**St. Louis encephalitis virus** (**SLEV**) (Table 2, Figure 2f) is common in the United States. The transmission cycle of the virus includes wild and domestic birds, and mosquitoes of the *Culex* genus as vectors [121,122,123,124]. **Murray Valley encephalitis virus** (**MVEV**) (Table 2, Figure 2e) is endemic to the territories of Australia and Oceania. The virus transmission cycle includes a variety of animals and waterfowl [125,126,127,128]. **West Nile virus** (**WNV**) (Table 2, Figure 2d) is transmitted by *Culex* spp. mosquito bites, and the reservoirs of the virus are mainly wild birds. The neuroinvasive form of the infection is the most severe form of the disease; more often, the infection in humans may be asymptomatic or manifest in a mild form [129,130,131,132].

**Kunjin virus** (**KUNV**) (Table 2, Figure 2e) is found in Australia and Oceania, like MVEV, severe manifestations of this rare infection can include encephalitis [133,134]. **Usutu virus** (**USUV**) (Table 2, Figure 2a) is distributed throughout the African continent, but has also been detected in European countries. The transmission cycle of the virus includes small insectivorous bats, rodents, and shrews; the virus is transmitted by *Culex annulirostris* mosquitoes [135,136,137,138].

**Zika virus** (**ZIKV**) (Table 2, Figure 2f) is transmitted to humans by *Aedes aegypti* and *Aedes albopictus* mosquito bites. For a long time, it was believed that the spread of the Zika virus was limited, but since 2015, the number of cases of this virus has sharply increased in a number of countries [139,140]. Outbreaks have been reported in Brazil and Central and North America. Infections were associated with significant increases in the incidence of microcephaly and Guillain–Barré syndrome in outbreak regions [139,140]. Evidence suggests that ZIKV may exhibit a higher affinity for placental cells compared to other flaviviruses [141]. Infants exposed to ZIKV during prenatal development, even without structural brain pathologies, may still experience neurological sequelae and developmental delays [142]. Currently, there is compelling evidence linking ZIKV to the aforementioned pathologies; however, further investigation of this phenomenon is warranted.

***Peribunyaviridae***. Representatives of the genus *Orthobunyavirus*, assigned to this family, include a wide range of arboviruses, including those transmitted by dipteran insects. This review included 17 species associated with CNS lesions. CNS diseases associated with orthobunyaviruses can be divided into two types: congenital and postnatal. In humans, the most common postnatal diseases of the central nervous system are meningitis and encephalitis [143]. Ortabunyaviruses are widely represented in endemic regions around the world, and are not only limited to regions with warm climates, i.e., they do not only refer to tropical pathogens [79,143].

**Bunyamwera virus** (**BUNV**) (Table 2, Figure 2c) was first isolated in 1943 from Aedes mosquitoes in Uganda [144,145]. In the 1950s, the virus was detected in KwaZulu-Natal in a sample from an adult male with severe headache, neck stiffness, and fever. Another study conducted in the same area revealed a 54% seropositivity rate among adults [144]. The predominant vectors of the spread are mosquitoes; however, Binder et al. reported the detection of BUNV in ixodid ticks [146]. Nowadays, several African countries are considered as endemic areas [145]; however, detections of the virus have also been reported outside Africa, in Brazil and Argentina [145,146]. In 2013, BUNV was first isolated from horses with neurological symptoms and a fatal outcome in Argentina [145]. In humans, BUNV predominantly causes mild illness characterized by febrile symptoms, and the onset of severe symptoms is common in children [145]. But in immunocompromised patients, BUNV infection can progress to encephalitis and meningitis [144,145]. Other mammals are also susceptible to infection, including domestic ruminants, in which infection results in severe symptoms such as spontaneous abortion and fetal defects [145]; antibodies to the virus have also been detected in a number of waterfowl in the KwaZulu-Natal province of South Africa [144].

**Germiston virus** (**GERV**) (Table 2, Figure 2c) is transmitted through the bites of *Culex* mosquitoes. In rare cases, infection is also possible through contact with infected tissues. The region of virus circulation is reportedly represented by Africa, in particular, the South Africa region. Complications of the disease after infection with this virus can manifest as meningoencephalitis and encephalitis [79,143,144,147].

**Xingu virus** (**XINV**) (Table 2, Figure 2g) was discovered in Brazil, and the natural vectors of the virus are mosquitoes (as for many other viruses in the *Orthobunyavirus* genus) [79,148]. Xingu is a member of the Bunyamwera serogroup [79,148]. The incidence of this virus is expected to be limited to very rare sporadic cases. A fatal case of Xingu disease was reported in which the patient presented with fever, headache, and jaundice [148]. However, the patient was also seropositive for hepatitis B, which in turn makes it difficult to remember the role of the virus in the development of the disease [148]. Additionally, there is a mention of the manifestation of infection with meningoencephalitis [79].

**Cache Valley virus** (**CVV**) (Table 2, Figure 2c) is reportedly circulating in several states in the United States; the main vectors of the virus are mosquitoes of the genus *Culex*. In humans, the manifestations of infection can include brain lesions (meningitis, meningoencephalitis, and encephalitis) [149,150,151,152].

**California encephalitis virus** (**CEV**) (Table 2, Figure 2c), **Jamestown Canyon virus** (**JEV**) (Table 2, Figure 2e), **Keystone virus** (**KEYV**) (Table 2, Figure 2e), **Main Drain virus** (**MDV**) (Table 2, Figure 2e), **snowshoe hare virus** (**SSHV**) Table 2, Figure 2b), **La Crosse virus** (**LACV**), (Table 2, Figure 2b) and **Tensaw virus** (**TENV**) (Table 2, Figure 2b) as well as Cache Valley virus, are also endemic to the United States and Canada. All of these viruses are transmitted to susceptible mammals through mosquito and midge (for Main Drain virus [66,143]) bites. However, for Tensaw virus, cases of intrauterine transmission have been documented, leading to the subsequent development of brain pathologies in the fetus. The clinical manifestations of infection with these viruses in humans include meningitis, encephalitis, and meningoencephalitis (see Table 2).

**Cristoli virus** (Table 2, Figure 2c), recently identified in France, was obtained from a patient with fatal encephalitis and an immunosuppressed status [153]. Mosquitoes are currently the suspected vectors of the virus [153]. Cristoli virus is closely related to Umbre virus, a member of the Turlock serogroup not previously associated with human disease [153]. The authors of the work [153] describing this clinical case suggested that Cristoli virus is endemic in France.

**Khatanga virus** (**KHATV**) (Table 2, Figure 2e) was found in the territory of the Russian Federation, in both the European part and Siberia. Amplification hosts include various wild sylvatic animals and, in some cases, domestic animals [79,154,155]. KHATV transmission occurs through the bites of blood-sucking insects of the genera *Aedes*, *Culiseta*, *Culex*, and *Anopheles*. Infection in humans may be accompanied, in particular, by encephalitis.

**Inkoo virus** (**INKV**) (Table 2, Figure 2e) is spread in the Russian Federation and Northern European countries, and transmitted by the bites of *Aedes* mosquitoes. In humans, INKV infection can cause encephalitis and other complications of the nervous system [52,79,156,157,158,159].

**Ťahyňa virus** (**TAHV**) (Table 2, Figure 2b,g) was found in Central Europe and China (Xinjiang, Qinghai, and Inner Mongolia). TAHV infection can cause meningitis, meningoencephalitis, encephalomyelitis, and encephalitis [79,160,161,162,163,164,165,166].

**Oropouche virus** (**OROV**) (Table 2, Figure 2g) is distributed in Latin America and is considered one of the most important arboviruses causing febrile illness in humans [167,168]. Reported cases of Oropouche fever have occurred in Brazil, Panama, Peru, and Trinidad and Tobago [167,168]. Oropouche fever occurs mainly during the rainy season, which is associated with an increase in the number of the main vectors of the virus belonging to the genera *Culex* and *Aedes* [167,168]. It is also noted that in addition to the spread of the virus between the vector and a susceptible host (pale-throated sloths, non-human primates, and birds) in the sylvatic cycle, the virus is well transmitted in the urban cycle between already infected people with the participation of the *Culicoides paraensis* vector [167,168]. There have been cases of CNS lesions occurring in patients; however, some of these patients already had concomitant diseases, including neurocysticercosis [168]. In total, during the entire observation period, fewer than 10 sporadic cases of central nervous system damage due to OROV infection were recorded in medical practice [168].

**Tucunduba virus** (**TUCV**) (Table 2, Figure 2a) and **Guaroa virus** (**GROV**) (Table 2, Figure 2a,b) are also common in Latin America. GROV transmission occurs through the bites of *Anopheles* mosquitoes [143,169], and TUCV is transmitted by mosquitoes of the genera *Culex*, *Wyeomia*, *Sabethes*, *Psorophora*, *Limatus*, and *Trichoprosopon* [170]. Complications in the nervous system can manifest as meningoencephalitis [170] and paresis [143,169].

**Shuni virus** (**SHUV**) (Table 2, Figure 2a) is transmitted by mosquitoes and Cullicoides midges; the virus is distributed in the territories of South Africa (Gauteng Province), Israel, and Nigeria; in some cases, complications of the disease may include meningitis and encephalitis [66,171].

**Ntwetwe virus** (**NTWV**) (Table 2, Figure 2e) and **Ilesha virus** (**ILEV**) (Table 2, Figure 2e) also circulate in Africa. The circulation area of **ILEV** includes the territories of Cameroon, Central African Republic, Nigeria, Senegal, Uganda, Madagascar, Ghana, and Niger [144,172]. Clinical manifestations of the disease in the nervous system can include meningitis [144,172]. **NTWV** was found in Uganda in patients with fatal encephalopathy and encephalitis [143,173].

**Umbre virus** (**UMBV**) (Table 2, Figure 2b,g) was first discovered in mosquitoes (*Culex* genus) in India in the 1950s [174]. Further research was able to detect viruses similar to UMBV in Australia, Malaysia, and the south of France [174]. Two clinical cases of patients with encephalitis and weakened immunity caused by UMBV (found in biomaterial) were reported, potentially exposing its neuroinvasive potential [174].

***Phenuiviridae.*** As reported above, representatives of this family are transmitted not only by ticks. Between mosquito- and sandfly-borne Phenuiviruses, the following pathogens are well known: **Rift Valley fever virus** (**RVFV**) and sandfly fever viruses (SFVs).

**Rift Valley fever virus** (**RVFV**) (Table 2, Figure 2b,f) is the causative agent of the febrile disease of the same name, most often found in African countries [139,175]. However, outside the African continent, cases have been reported in Europe, Asia, and the USA [139]. The main route of transmission of the virus to humans is through the bites of *Culex* mosquitoes; the transmission of the virus is also possible through contact with the blood and other biological material of infected domestic animals, for example, cows, goats, and buffalo [139]. The direct transmission (human-to-human) of the virus has not been recorded [139]. In animals, the virus causes severe disease, while in most humans, it is either asymptomatic or has mild febrile manifestations [139]. However, in approximately 10% of patients, the disease may present with bleeding and encephalitis [139].

The **Sandfly fever** group of viruses includes sandfly fever Sicilian virus (SFSV), sandfly fever Naples virus (SFNV), and **Toscana virus** (**TOSV**) (Table 2, Figure 2a), associated with human febrile diseases. The endemic region of distribution of these viruses includes Italy, France, Spain, Slovenia, Turkey, Portugal, and Greece [176,177,178]. In other countries, such as Sweden, cases of infection have also been reported. The transmission of these viruses occurs through the bites of the virus-competent phlebotomine midges *Phlebotomus perniciosus* and *Phlebotomus perfiliewi* [176,177,178]. In humans, the infection mainly causes a mild febrile illness with neurological damage (aseptic meningitis or encephalitis), sometimes with complete recovery. Other common symptoms are leukopenia, neck stiffness, a decreased level of consciousness, tremors, and paresis [176,177,178].

***Rhabdoviridae.* Chandipura virus** (**CHPV**) (Table 2, Figure 2b,c) is an important cause of morbidity, primarily in India. However, apart from India, to date, CHPV has also been detected in Bhutan, Nepal, Sri Lanka, and African countries (Nigeria and Senegal) [179]. The virus was first discovered during a fever outbreak in 1965 in Nagpur, Maharashtra. The transmission cycle of CHPV involves mosquitoes, which are thought to carry the virus continuously in endemic regions [179]. The presence of neutralizing antibodies to CHPV in the blood of pigs, buffalo, cattle, goats, and sheep indicates the constant circulation of the virus in the regions of distribution. CHPV is the causative agent of acute encephalitis, especially among children under 15 years of age [179]. A critical feature of the infection is the sudden onset of clinical symptoms, including neurological complications (within 24 h) and a high mortality [179].

***Sedoreoviridae.*** Arboviruses **Orungo virus** (**ORUV**) and **Banna virus** (**BAV**) are rare causative agents of febrile diseases, and, in some cases, with complications in the nervous system. **Orungo virus** (**ORUV**) (Table 2, Figure 2g) is transmitted by *Aedes* spp., *Culex* spp., and *Anopheles* spp. in regions of sub-Saharan Africa. Manifestations of infection in humans include acute fever and headaches. One case of encephalitis in a child, with convulsions and flaccid paralysis, has also been reported [88]. For **Banna virus** (**BAV**) (Table 2, Figure 2b,c), the vectors are *Culex* spp. and presumably other types of mosquitoes. There are reports in the literature of BAV infection in humans, manifested in flu-like symptoms, myalgia, fever, and encephalitis [180].

***Togaviridae***. Many important pathogens of humans and animals are included in the genus *Alphavirus*, including viruses transmitted by mosquito bites: Eastern equine encephalitis virus North American (EEEV-NA), Venezuelan equine encephalitis virus (VEEV), Western equine encephalitis virus (WEEV), and Chikungunya virus (CHIKV). **Madariaga virus** (**MADV**) (Table 2, Figure 2b,e) [181,182,183,184], **Mayaro virus** (**MAYV**) (Table 2, Figure 2e) [103,185,186], **Middelburg virus** (**MIDV**) (Table 2, Figure 2e) [187,188], **Ross River virus** (**RRV**) (Table 2, Figure 2b,g) [189,190], **Sindbis virus** (**SINV**) (Table 2, Figure 2g) [70,158,159,181,191], and **Tonate virus** (**TONV**) (Table 2, Figure 2a) [192,193] are recognized as relatively rare but important causative agents of neuroinfections in humans, including encephalitis and aseptic meningitis. The geographic distribution of these viruses is currently limited to the tropical regions of South and Central America, with the exception of RRV (New Guinea and Australia), MIDV (South Africa and Zimbabwe), and SINV. The latter has been repeatedly discovered in various regions of the globe with very different climatic conditions: in African countries, Australia, Europe, Russia, and Asian countries.

**Chikungunya virus** (**CHIKV**) (Table 2, Figure 2f) was first described after a series of outbreaks in India and South Asia in the 1960s. The virus is endemic to tropical and subtropical regions. The transmission of the virus is mainly carried out by *Aedes aegypti* and *Aedes albopictus* [129,139]. Chikungunya fever, in addition to cases where complications in the nervous system are observed, such as encephalitis and convulsions, is also manifested in a rash, headache, and severe polyarthralgia [129,139].

**Eastern equine encephalitis virus North American** (**EEEV-NA**) (Table 2, Figure 2b,c), **Venezuelan equine encephalitis virus** (**VEEV**) (Table 2, Figure 2f), and **Western equine encephalitis virus** (**WEEV**) (Table 2, Figure 2e) are distributed in South and North America. All three viruses actively circulate in the vector cycle, which includes wild birds, rodents, and mosquitoes. Transmission to humans occurs through *Culex* and *Aedes* mosquito bites. Although the disease caused by these viruses is self-limiting, neurological complications can include severe encephalitis, with a mortality rate of 30 to 75% in humans.

**Table 2:** List of mosquito- and midge-borne viral agents of neuroinflammation.

Family	Genus	Species	Common Names or Subspecies/ Acronym(s)	Genome	Host-Source, Vector, Transmission	Geographic Distribution	NS Pathology	Reference
		(Sub)Species complex/ Acronym(s)						
* Flaviviridae *	* Orthoflavivirus *	* Orthoflavivirus * *aroaense*	Bussuquara virus/ BSQV	ssRNA(+)	Hs: Non-human primates, rodents, wild birds V: Mosquitoes (*Culex* spp.) T: With mosquito bites	Brazil (Pará state), Panama	Encephalitis	[110,129]
			Iguape virus/ IGUV	ssRNA(+)	Hs: Wild birds V: Mosquitoes (*Aedes* spp.) T: With mosquito bites	Brazil (Sao Paulo state)	Encephalitis	[109,110,194]
		* Orthoflavivirus * *cacipacoreense*	Cacipacoré virus/ CPCV	ssRNA(+)	Hs: Wild birds (*Formicarius analis*) V: Mosquitoes (*Culex* spp.) T: With mosquito bites	Brazil (Pará and Rondônia states), Amazon region	Encephalitis	[129]
		* Orthoflavivirus * *denguei*	Dengue virus/ DENV	ssRNA(+)	Hs: Non-human primates (macaques—*Macaca* spp.; Surilis—*Presbytis* spp.) V: Mosquitoes (*Aedes aegypti*, *Aedes albopictus, Aedes scutellaris*, *Aedes polynesiensis*; *Aedes furcifer*, *Aedes vittatus*, *Aedes tailori*, *Aedes luteocephalus*—equatorial parts of Africa) T: With mosquito bites; Human-to-human contact (breastfeeding); Congenital infection.	Focally worldwide spread; High treat: Africa (Sudan, Egypt, Eritrea, Djibouti, Ethiopia, Kenya, Somalia, Tanzania, Mauritius, Mozambique, Seychelles, Angola, Cameroon, Burkina Faso, Côte d’Ivoire, Senegal); the Caribbean basin, Central America, South America, southeastern Asia, Oceania	Encephalitis, meningitis, meningoencephalitis, encephalomyelitis, acute cerebellitis, polyneuritis, encephalopathy, Parkinsonian symptoms	[79,195,196,197,198,199,200,201,202]
		* Orthoflavivirus * *flavi*	Yellow fever virus/ YFV	ssRNA(+)	Hs: Non-human primates V: Mosquitoes (*Aedes* spp., *Haemagogus* spp., *Sabethes* spp.); ticks (*Amblyomma variegatum*)—in Africa, extremely rare T: With mosquito bites; With tick bites.	Endemic regions: West Africa (Benin, Burkina Faso, Cape Verde, Côte d’Ivoire, Equatorial Guinea, Gambia, Ghana, Guinea, Guinea-Bissau, Liberia, Mali, Mauritania, Niger, Nigeria, Sao Tome and Principe, Senegal, Sierra Leone, Togo); Central Africa (Angola, Burundi, Cameroon, Central African Republic, Chad, Democratic Republic of the Congo, Gabon, Rwanda); East Africa (Ethiopia, Kenya, Somalia, Sudan, Tanzania, Uganda); Panama; South America (Argentina, Bolivia, Brazil, Colombia, Ecuador, Guyana, French Guyana, Paraguay, Peru, Suriname, Trinidad and Tobago, Venezuela)	Encephalitis, YFV vaccine-associated encephalitis, ADEM, Guillain–Barré syndrome, meningitis, meningoencephalitis	[112,113,114,115,116,117,118]
		* Orthoflavivirus * *ilheusense*	Ilhéus virus/ ILHV	ssRNA(+)	Hs: Wild birds V: Mosquitoes (*Aedes* spp., *Psorophora* spp.) T: With mosquito bites	Brazil (Pará and São Paulo states, Pantanal region)	Encephalitis	[129]
			Rocio virus/ ROCV	ssRNA(+)	Hs: Rufous-collared sparrow (*Zonotrichia capensis*) V: Mosquitoes (*Ochleratus* spp., *Psorofora ferox*; specific antibodies: double-collared seedeater (*Sporophila caerulescen*); creamy-bellied thrush (*Turdus amaurochalinus*); equines; water buffalo (*Bubalus bubalis*); marsupials T: With mosquito bites	Southeast Brazil (Sao Paulo state—endemic region); Other regions of virus circulation: Goiás state, Rio de Janeiro state, Mato Grosso do Sul state, Paraíba state, Mato Grosso state	Encephalitis, meningoencephalitis, meningitis	[119,133,203]
		* Orthoflavivirus * *japonicum*	Japanese encephalitis virus/ JEV	ssRNA(+)	Hs: Wild aquatic birds, domestic birds, domestic pigs V: Mosquitoes (Main vectors*—Culex tritaeniorhynchus*, *Culex vishnui, Culex gelidus*) T: With mosquito bites	Focally worldwide spread; Asia–Pacific region, Southeast Asia, Australia	Encephalitis, meningoencephalitis, meningitis	[120,204,205,206,207,208]
		* Orthoflavivirus * *louisense*	St. Louis encephalitis virus/ SLEV	ssRNA(+)	Hs: Sylvatic, peridomestic, and urban birds (sparrows—*Passer* sp.; pigeons—*Columba* sp.; blue jay—*Cyanocitta cristata*; robins—*Turdus* sp.) V: Mosquitoes (*Culex tarsalis*, *Culex pipiens*, *Culex quinquefasciatus*) T: With mosquito bites	United States (Eastern and Central states)	Encephalitis, meningoencephalitis, meningitis	[121,122,123,124]
		* Orthoflavivirus * *murrayense*	Murray Valley encephalitis virus/ MVEV	ssRNA(+)	Hs: Wild animals: marsupials (kangaroos; agile wallabies—*Notamacropus agilis*); rabbits (*Leporidae*)*;* rodents; wild birds—Galahs (*Cacatuidae*); water birds (rufous night heron—*Nycticorax caledonicus*; Pacific black duck—*Anas superciliosa*); domestic animals and birds V: Mosquitoes (*Culex annulirostris*, *Culex sitiens*) T: With mosquito bites	Australia (Western Australia, Northern Territory, New South Wales, Victoria); Papua New Guinea; Indonesia; Canada (Alberta)—imported infection	Encephalitis	[125,126,127,128]
		* Orthoflavivirus * *nilense*	West Nile virus/ WNV	ssRNA(+)	Hs: Wild birds, domestic animals (horses, sheep), alligators, lake frog (*Rana ridibunda*—competent reservoir (Russia)) V: Mosquitoed (*Culex* spp.) T: With mosquito bites; Human-to-human transmission (organ transplantation, blood transfusion, placental route)	Endemic region: East Africa (Uganda) Many cases: North America, Brazil, Middle East, Europe, Asia, Regions of Africa Worldwide spread	Meningitis, encephalitis, poliomyelitis	[129,130,131,132,209,210]
			Kunjin virus/ KUNV	ssRNA(+)	Hs: Wild birds, domestic animals (horses, sheep), alligators V: Mosquitoes (*Culex annulirostris*) T: With mosquito bites	Australia (tropical north regions), Oceania	Encephalitis	[133,134]
		* Orthoflavivirus * *usutuense*	Usutu virus/ USUV	ssRNA(+)	Hs: Wild passerine birds, insectivorous microbats (*Pipistrellus* sp.), equines, rodents, shrews V: Mosquitoes (*Culex* spp., *Aedes* spp., *Mansonia* spp., *Anopheles* spp.) T: With mosquito bites	Africa (South Africa, Central African Republic, Senegal, Côte d’Ivoire, Nigeria, Uganda, Burkina Faso, Tunisia, Morocco); Europe (introductions: France, Germany, Italy, Austria, Serbia)	Encephalitis, meningoencephalitis	[135,136,137,138]
		* Orthoflavivirus * *zikaense*	Zika virus/ ZIKV	ssRNA(+)	Hs: Non-human primates V: Mosquitoes (*Aedes* spp.) T: With mosquito bites; Human-to-human transmission (organ transplantation, blood transfusion, placental route); Contact with infected fomites.	Brazil, Central and North America	Guillain–Barré syndrome, fetal microcephaly, myelitis, meningoencephalitis	[139,140,141,211]
* Peribunyaviridae *	* Orthobunyavirus *	* Orthobunyavirus * *bunyamweraense*	Bunyamwera virus/ BUNV	ssRNA(−)	Hs: Wild waterfowls V: Mosquitoes (*Aedes circumluteolus*); hard ticks (*Amblyomma dubitatum*, *Amblyomma sculptum*)—presumably T: With mosquito bites; With tick bites—presumably	Uganda, Tanzania, Mozambique, Nigeria, Guinea, South Africa (KwaZulu-Natal province), Democratic Republic of Congo, Botswana, Namibia (Caprivi region), Senegal, Ivory Cost, Cameroon, Central African Republic, Kenya, Madagascar; Argentina, Brazil (Minas Gerais (ticks))	Encephalitis, meningitis	[144,145,146]
			Germiston virus/ GERV	ssRNA(−)	Hs: Wild animals (virus isolation); domestic animals (antibody detection) V: Mosquitoes (*Culex theileri*, *Culex rubinotus*) T: With mosquito bites; Direct contact with infected tissue and fomites	Africa; South Africa region	Encephalitis, meningoencephalitis (both sporadic cases; laboratory work infection); mental confusion	[79,143,144,147]
			Xingu virus/ XINV	ssRNA(−)	Hs: Not identified V: Mosquitoes T: With mosquito bites—presumably	South America (Brazil)	Encephalitis/meningoencephalitis (both sporadic cases)	[79,148]
		* Orthobunyavirus * *cacheense*	Cache Valley virus/ CVV	ssRNA(−)	Hs: Domestic ruminants (equines, cattle); deer V: Mosquitoes (*Culex* spp.) T: With mosquito bites	US (Utah, North Carolina, Missouri, Wisconsin, New York)	Encephalitis, meningoencephalitis, meningitis	[149,150,151,152]
		* Cristoli virus * ^1^	–	ssRNA(−)	Hs: Not identified V: Mosquito—presumably T: With mosquito bites—presumably	France (Île-de-France region, including Paris)	Encephalitis	[153]
		* Orthobunyavirus * *encephalitidis*	California encephalitis virus/ CEV	ssRNA(−)	Hs: Equines V: Mosquitoes T: With mosquito bites	US (California)	Encephalitis	[79,212]
		* Orthobunyavirus * *guaroaense*	Guaroa virus/ GROV	ssRNA(−)	Hs: Mosquitoes (*Anopheles* (*Kerteszia*) *neivai*) V: Mosquitoes—presumably T: With mosquito bites—presumably	Brazil, Colombia, Panama, Bolivia	Paresis	[143,169]
		* Orthobunyavirus * *ileshaense*	Ilesha virus/ ILEV	ssRNA(−)	Hs: – V: Mosquitoes (*Anopheles gambiae*) T: With mosquito bites	Cameroon, Central African Republic, Nigeria, Senegal, Uganda; Madagascar (virus isolation from infected persons); Ghana and Niger (antibody from infected persons)	Meningoencephalitis	[144,172]
		* Orthobunyavirus * *jamestownense*	Jamestown Canyon virus/ JCV	ssRNA(−)	Hs: White-tailed deer (*Odocoileus virginianus*), moose (*Alces alces*), elk (*Cervus elaphus*), bison (*Bison bison*) V: Mosquitoes (*Culiseta inornata*, *Aedes* spp., *Anopheles* spp.) T: With mosquito bites	US (Minnesota, Wisconsin); Canada (British Columbia, Alberta, Saskatchewan, Manitoba, Ontario, Quebec, New Brunswick, Nova Scotia)	Encephalitis, meningoencephalitis, meningitis	[79,185,186,213,214]
			Inkoo virus/ INKV	ssRNA(−)	Hs: Wild birds V: Mosquitoes (*Aedes* spp.) T: With mosquito bites	Finland, Sweden, Norway, Russia	Asthenoneurologic disturbances, microfocal neurologic symptoms, encephalitis	[52,79,156,157,158,159]
		* Orthobunyavirus * *kernense*	Main Drain virus/ MDV	ssRNA(−)	Hs: Horses, wild birds, black-tailed jackrabbit (*Lepus californicus*) V: Mosquitoes (*Culicidae*)—occasional vector; biting midges (*Ceratopogonidae, Culicoides variipennis*) T: With mosquito or midge bites	US (California)	Unspecified CNS disease	[66,143]
		* Orthobunyavirus * *keystoneense*	Keystone virus/ KEYV	ssRNA(−)	Hs: Squirrels, raccoons, whitetail deer (*Odocoileus virginianus*) V: Mosquitoes (*Aedes* spp.) T: With mosquito bites	US (Florida, coastal regions of the Chesapeake Bay)	Encephalitis, meningitis	[191,215]
		* Orthobunyavirus * *khatangaense*	Snowshoe hare virus/ SSHV	ssRNA(−)	Hs: Hares, squirrels V: Mosquitoes (*Aedes* spp.) T: With mosquito bites	US, Canada	Meningoencephalitis, encephalitis, meningitis	[79,216,217,218,219]
			Khatanga virus; Chatanga virus/ KHATV	ssRNA(−)	Hs: Wild sylvatic animals; domestic animals—presumably V: Mosquitoes (*Aedes* spp., *Culiseta* spp., *Culex* spp., *Anopheles* spp.) T: With mosquito bites	Russia (European part, western, middle and northeastern Siberia)	Encephalitis	[79,154,155]
		* Orthobunyavirus * *lacrosseense*	La Crosse virus/ LACV	ssRNA(−)	Hs: Chipmunks, squirrels V: Mosquitoes (*Aedes* spp.) T: With mosquito bites	US (Ohio, Wisconsin, Minnesota, Indiana, Illinois, Iowa, North Carolina, Tennessee, West Virginia, Georgia, Virginia, Kentucky, Rhode Island)	Encephalitis	[79,134,139,140]
		* Orthobunyavirus oropoucheense *	Oropouche virus/ OROV	ssRNA(−)	Hs: Pale-throated sloths, non-human primates V: Mosquitoes (*Culex* spp., *Aedes* spp.); biting midges (*Culicoides*) T: With mosquito bites	Brazil, Panama, Peru, Trinidad and Tobago	Meningitis	[167,168,171]
		* Orthobunyavirus shuniense *	Shuni virus/ SHUV	ssRNA(−)	Hs: Horses, domestic cattle V: Mosquitoes (*Culex theileri*); *Cullicoides* midges T: With mosquito and midge bites	South Africa (Gauteng province), Israel, Nigeria	Encephalitis, meningitis	[66,171]
		* Orthobunyavirus * *tahynaense*	Ťahyňa virus/ TAHV	ssRNA(−)	Hs: Small wild mammals V: Mosquitoes (*Culex* spp., *Aedes* spp.) T: With mosquito bites	Central Europe; China (Xinjiang, Qinghai, Inner Mongolia)	Meningitis, meningoencephalitis, encephalomyelitis, encephalitis	[79,160,161,162,163,164,165,166]
		* Orthobunyavirus * *tensawense*	Tensaw virus/ TENV	ssRNA(−)	Hs: Sylvatic rodents, foxes, raccoons, dogs, cows V: Mosquitoes (*Aedes vexans*, *Anopheles crucians*, *Coquillettidia perturbans*, *Culex salinarius*, *Uranotaenia sapphirina*) T: With mosquito bites—presumably; Congenital infection.	US (Alabama, Florida)	Rabies-like symptoms, encephalitis, micro-/macrocephaly	[143,220]
		* Orthobunyavirus umbreense *	Umbre virus/ UMBV	ssRNA(−)	Hs: Not identified V: Mosquitoes (*Culex* spp.) T: With mosquito bites—presumably	India, Australia (Queensland—Umbre-related viruses); Malaysia (Umbre-related domestic avian pathogenic virus); France—presumably	Lethal encephalitis	[174]
		* Orthobunyavirus * *wyeomyiae*	Tucunduba virus/ TUCV	ssRNA(−)	Hs: – V: Mosquitoes (*Culex* spp., *Wyeomia* spp., *Sabethes* spp., *Psorophora* spp., *Limatus* spp., *Trichoprosopon* spp.) T: With mosquito bites	Brazil	Meningoencephalitis	[170]
		* Ntwetwe virus * ^1^	Ntwetwe virus ^1^/ NTWV ^1^	ssRNA(−)	Hs: – V: Mosquitoes (*Anopheles* spp.) T: –	Uganda	Fatal encephalopathy, encephalitis	[143,173]
* Phenuiviridae *	* Phlebovirus *	* Phlebovirus * *riftense*	Rift Valley Fever virus/ RVFV	ssRNA(+/−)	Hs: Wild and domestic animals V: Mosquitoes (*Culex* spp.) T: With mosquito bites; Direct contact with contaminated biological fluids.	Kenya, Tanzania, South Africa, Sudan, Egypt, Madagascar, Somalia, Mauritania, Botswana, Namibia	Meningoencephalitis, encephalitis	[139,175]
		* Phlebovirus * *toscanaense*	Toscana virus/ TOSV	ssRNA(+/−)	Hs: – V: Sandflies (*Phlebotomus perniciosus*, *Phlebotomus perfiliewi*) T: With sandfly bites	Italy, Spain, Slovenia, Turkey, Portugal, Greece, Cyprus, Southern France, the Balkans, the Black Sea coast, Iraq, Iran, Pakistan, Afghanistan, India	Meningitis, meningoencephalitis	[176,177,178]
* Rhabdoviridae *	* Vesiculovirus *	* Vesiculovirus * *chandipura*	Chandipura virus/ CHPV	ssRNA(−)	Hs: Pigs, buffalo, cattle V: Mosquitoes (*Phlebotomus* spp.) T: With mosquito bites	India, Bhutan, Nepal, Sri Lanka, Nigeria, Senegal	Encephalitis	[179]
* Sedoreoviridae *	* Orbivirus *	* Orungo virus *	Orungo virus/ ORUV	dsRNA	Hs: – V: Mosquito (*Aedes* spp., *Culex* spp., *Anopheles* spp.) T: With mosquito bites	Regions of sub-Saharan Africa	Encephalitis	[88]
	* Seadornavirus *	* Banna virus *	Banna virus/ BAV	dsRNA	Hs: Domestic pigs, cattle V: Mosquitoes (*Culex tritaeniorhynchus*, *Culex pipiens pallens*, *Culex annulus, Culex pseudovishnui*, *Culex modestus, Anopheles sinensis*, *Aedes vagus*, *Aedes albopictus*, *Aedes vexans*, *Aedes dorsalis*); Midges (*Culicoides* sp.) T: With mosquito bites	Indonesia, China, Vietnam	Encephalitis	[180,221,222]
* Togaviridae *	* Alphavirus *	* Chikungunya * *virus*	Chikungunya virus/ CHIKV	ssRNA(+)	V: Mosquitoes (*Aedes* spp.) T: With mosquito bites	Africa, Southeastern Asia, Europe (imported infection), North America	Myelitis, encephalitis	[129,139,223]
		* Eastern equine * *encephalitis virus*	Eastern equine encephalitis virus North American/ EEEV-NA	ssRNA(+)	Hs: Birds, mammals V: Mosquitoes (*Aedes* spp., *Culex* spp., *Anopheles* spp.) T: With mosquito bites	North America (Massachusetts, Michigan, Florida, Georgia, North Carolina), the Caribbean region	Encephalitis	[224,225,226]
		* Madariaga virus *	Madariaga virus; Eastern equine encephalitis virus South American/ MADV; EEEV-SA	ssRNA(+)	Hs: Birds, mammals V: Mosquitoes (*Aedes* spp., *Culex* spp., *Anopheles* spp.) T: With mosquito bites	South America (Panama, Venezuela), Haiti	Encephalitis, encephalomyelitis	[181,182,183,184]
		* Mayaro virus *	Mayaro virus/ MAYV	ssRNA(+)	Hs: Non-human primates, migratory birds V: Mosquitoes (*Haemagogus* spp.—particularly *Haemagogus janthinomys*); *Culex* spp., *Mansonia* spp., *Aedes* spp., *Psorophora* spp., *Sabethes* spp. T: With mosquito bites	Europe (Germany, France, Netherlands, Switzerland—imported infection); United states (isolated in non-human primates, migratory birds); Mexico, Trinidad and Tobago, Brazil, Surinam, French Guiana, Venezuela, Haiti, Bolivia, Peru, Ecuador, Colombia (isolated from mosquitoes)	Encephalopathy	[129,227,228]
		* Middelburg virus *	Middelburg virus/ MIDV	ssRNA(+)	Hs: Equines, mice, sheep V: Mosquitoes (*Aedes* spp.) T: With mosquito bites	South Africa, Zimbabwe	Meningo-encephalitis	[187,188]
		* Ross River virus *	Ross River virus/ RRV	ssRNA(+)	Hs: Mammals, birds V: Mosquitoes (*Culex* spp.) T: With mosquito bites	Australia, Papua New Guinea	Meningitis (rare cases); encephalitis (rare cases)	[189,190]
		* Sindbis virus *	Sindbis virus/ SINV	ssRNA(+)	Hs: Wild birds (*Corvus corone sardonius*—hooded crow); rodents; domestic animals V: Mosquitoes (*Culex* spp., *Anopheles* spp., *Coquillettidia* spp., *Aedes* spp., *Ocheleratus* spp.); Gamasidae ticks (*Ornithonyssus bacoti*), Ixodidae ticks (*Hyalomma marginatum*) T: With arthropod-vector bites	Africa (endemic regions—Egypt, South Africa, Uganda, Central African Republic, Sudan, Nigeria, and Zimbabwe), Europe (Germany, Sweden, Finland, Italy, Slovakia), Russia, the Middle East, the Philippines, Turkey, Azerbaijan, Israel, India, China, Malaysia, Australia (north regions), New Zealand	Meningitis—presumably	[66,79,192,229,230]
		* Tonate virus *	Tonate virus; Venezuelan equine encephalitis virus IIIB/ TONV; VEEV-IIIB	ssRNA(+)	Hs: Wild birds (*Psarocolius decumanus*—crested oropendola) V: Mosquitoes (*Culex portesi*) T: With mosquito bites	North America, South America (Surinam, French Guiana), Central America	Encephalitis	[192,193]
		* Venezuelan equine * *encephalitis virus*	Venezuelan equine encephalitis virus/ VEEV	ssRNA(+)	Hs: Wild rodents (cotton mouse—*Peromyscus gossypinus*; hispid cotton rat—*Sigmodon hispidus*; spiny rats—*Proechimys* spp.; *Oryzomys* spp., *Zigodontomys* spp., *Heteromys* spp.), equines, canids, pigs, wild birds, bats V: Mosquitoes (*Culex* spp., *Mansonia* spp., *Anopheles* spp., *Aedes* spp., *Psorophora* spp., *Sabethes* spp., *Haemagogus* spp., *Deinocerites* spp.); *Ochlerotatus taeniorhynchus* T: With mosquito bites	Costa Rica, Venezuela, Colombia, Belize, Peru, Ecuador, British Guyana, Guatemala, Argentina, Panama, Trinidad, Honduras, El Salvador, Nicaragua, Mexico; United States (Texas, Florida)	Encephalitis, meningitis	[15,52,66,192,231,232,233]
		* Western equine * *encephalitis virus*	Western equine encephalitis virus/ WEEV	ssRNA(+)	Hs: Wild birds (passerine); wild rodents, horses V: Mosquitoes (*Culex tarsalis*; *Aedes* spp.); *Ochlerotatus melanimon* (California), *Aedes dorsalis* (Utah, New Mexico), *Aedes campestris* (New Mexico) T: With mosquito bites	Brazil, Colombia, United States	Encephalitis, meningitis, encephalomyelitis	[15,129,233,234]

Taxonomic and trivial names are given according to the reports of the International Committee on Taxonomy of Viruses (ICTV) https://ictv.global/msl and https://ictv.global/vmr (versions from 2022/2023); ^1^ Commonly accepted alternative names not given in the reports of the International Committee on Taxonomy of Viruses (ICTV) (see above).

## 3. Zoonotic Viral Neuropathogens

There are an estimated 2.5 billion cases and 2.7 million deaths associated with zoonotic infections worldwide each year [235]. By some estimates, more than 60% of currently known pathogens affecting humans, and 75% of emerging diseases, are zoonotic [236,237,238]. Of the just over 500 known zoonotic viruses transmitted directly or indirectly, 120 have been identified as capable of causing human disease [236]. It is believed that 10,000 of the 40,000 viruses carried by mammals have zoonotic potential and may pose a threat as causes of epidemics [239].

Among all agents that cause zoonotic diseases, viruses are the most common [133], accounting for more than 65% of pathogens discovered since 1980 [240]. Most of these viruses are RNA-containing, which explains their high variability/lability, and as a result, their ability to exist in a wide range of hosts (unlike DNA viruses, which have greater genetic stability, limiting their range to a range of closely related animal hosts) [133,237]. The most effective carriers of viruses with zoonotic potential among vertebrates are bats and rodents. For example, rodents are reservoirs of about 80 zoonotic viruses [239].

Often, zoonotic viruses are low-pathogenic for their natural hosts (amplification or reservoir hosts), but when the virus enters a new and often “dead-end” host organism, they can manifest themselves as highly virulent agents [241]. It is assumed that, in some cases, the severity and lethality of the pathological process when affected by zoonotic viruses is primarily associated with the immune response of the new host [241].

***Arenaviridae***. Among the pathogens of this family, viruses of the ***Mammarenavirus*** genus are dangerous to humans. Representatives of the genus that are pathogenic for humans, with serious manifestations of infection (though asymptomatic cases have also been noted), most often cause hemorrhagic fever, including in immunocompetent individuals [239,242,243,244]. For example, endemic to regions of West Africa, *Mammarenavirus lassaense* (Lassa virus, LASV) causes Lassa fever, with a fatality rate that can reach about 69%, and is estimated to infect about 300,000 people annually [242,244].

Mammarenaviruses are divided into Old and New World viruses (the same division is used for Hantaviruses), based on their genetic relationship, geographic distribution, and epidemiological characteristics [243]. The spread of mammarenaviruses is closely related to their reservoir hosts, the vast majority of which belong to the order of rodents, including synanthropic and ubiquitous ones, such as house mice (*Mus musculus*) [245]. Among other mammals that are reservoirs of these mammarenaviruses, hedgehogs, jerboas, and some arthropods are also noted [246].

The main routes of transmission of viruses from a chronic animal carrier to a dead-end host, a human, are contact with fomites of infected animals and biological fluids, including through airborne droplets; when eating meat; or through trauma—as a result of bites or other damage to the skin [239].

Among the mammarenaviruses that can cause CNS pathologies in humans is **Lymphocytic choriomeningitis virus** (**LCMV)** (Table 3). LCMV is a relatively rare but clinically important pathogen (less than 0.5% of viral meningitis cases) [244,247]. The clinical spectrum of acquired LCMV infection is quite wide, from asymptomatic manifestations to those requiring serious medical intervention. In a third of patients, the disease is asymptomatic, but this does not apply to people with compromised immunity nor to cases of perinatal infection [49]. Severe manifestations of congenital infection in humans are meningitis, encephalitis, hydrocephalus, transverse myelitis, cerebellar hypoplasia, focal brain destruction, gyral dysplasia, and fetal death [49]. LCMV is the only known endemic mammarenavirus in Europe [246]. Just like other members of the genus, LCMV circulates within the rodent population, including *Mus musculus*, where the transmission of the virus from one generation to another occurs, including transplacentally; about 9% of the mouse population are chronic carriers of the virus [49,244,247]. Passive carriage by rodent reservoirs is possible since the virus is not cytolytic [49,247]. A subsequent release of the virus into the environment from a colonized reservoir organism is observed throughout the life of rodents, and does not depend on seasonal factors [49,247]. However, the risk of infection with lymphocytic choriomeningitis virus through contact with animals increases during periods of reproduction and an increase in the number of rodents. The transmission of the virus is possible in several ways: postnatally, through the transplantation of donor organs; upon contact with maternal secretions or blood during maternal viremia (rare cases); and perinatally, i.e., transplacentally [49,244,247].

**Figure 3:**
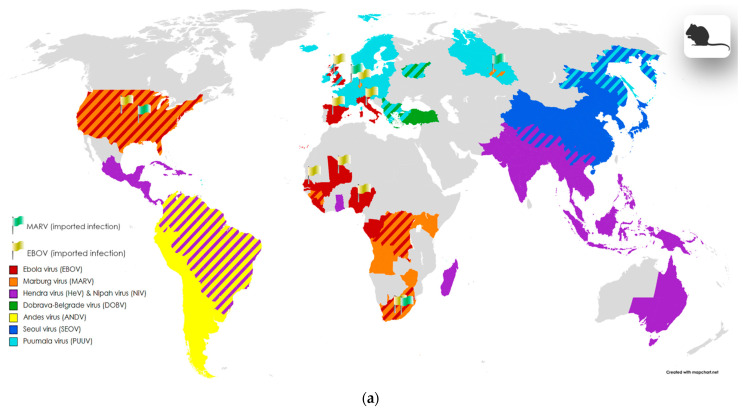

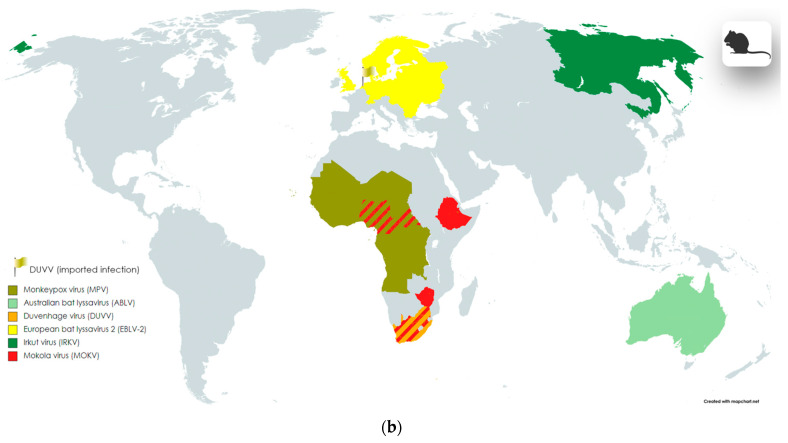
Geographic distribution of zoonotic (vertebrate) infections, including areas of endemic and imported cases involving nervous system symptoms. The division into blocks (**a**,**b**) was implemented to enhance the visualization of data on the maps; information about the taxonomic groups located on each block is provided in the legend within the pictures, as well as in the main text alongside the first mention of the corresponding virus; imported cases are marked with flags. Maps were created with the Map Chart online map-making tool (https://www.mapchart.net/index.html, accessed on 7 May 2024); graphic objects were created with the BioRender online science illustration making tool (https://www.biorender.com/, accessed on 7 May 2024). The images may contain slight inaccuracies in depicting the boundaries of virus distribution areas due to the limitation of the map scale and the choice of scientific sources utilized to create these illustrations.

***Filoviridae***. **Ebola virus** (**EBOV**) (Table 3, Figure 3a) is the causative agent of a severe form of viral hemorrhagic fever in humans. Outbreaks associated with the Ebola virus pose a serious threat to public health due to high case fatality rates of approximately 50% (previously, this ranged from 25 to 90%) [240,248]. The Ebola virus is endemic on the African continent, but there is a risk of possible spread to other territories. EBOV is an initially zoonotic infection that emerged sporadically in the human population and subsequently became transmitted from person to person [248]. The initial reservoirs of the virus are bats and non-human primates [248].

**Marburg virus** (**MARV**) (Table 3, Figure 3a), belonging to the same genus and family as the Ebola virus, is also common among bats and some primates [249]. The clinical manifestations of infection caused by the Marburg virus are similar to those of the Ebola virus: in addition to the symptoms of neuroinfections, hemorrhagic fever, liver failure, and the infection of the spleen and kidney tissues are observed. MARV has been the cause of several outbreaks since its simultaneous discovery and description in 1967 in Marburg, Frankfurt, and Belgrade. However, most MARV outbreaks have occurred in Africa [250].

***Hantaviridae***. Members of the family contain segmented negative-sense RNA genomes packaged in enveloped virions [251]. Hantavirus infections are natural focal zoonoses, predominantly associated with rodents [252,253]. Old World hantaviruses cause hemorrhagic fever with renal syndrome (HFRS) and are more common in Asia and Europe, while New World hantaviruses are found in the Americas and cause HPS (hemorrhagic pulmonary syndrome). HFRS, caused by Hantaan virus, **Dobrava** (**DOBV**) (Table 3, Figure 3a), Saaremaa virus, **Seoul virus** (**SEOV**) (Table 3, Figure 3a), and **Puumala virus** (**PUUV**) (Table 3, Figure 3a), may have a mortality rate of up to 15%, while HPS, caused by Sin Nombre virus (SNV) and **Andes virus** (**ANDV**) (Table 3, Figure 3a), has a mortality rate of up to 40% [254]. In Russia, hantaviruses are among the most common zoonoses [252,253]. Rodents serve as the natural reservoir for almost all known hantaviruses. In Eurasia, transmission is mainly associated with mice, rats, and voles [253]. Like the lymphocytic choriomeningitis virus, the prevalence of hantaviruses is ubiquitous and is associated with the habitats of certain groups of rodents (reservoirs), but some of the representatives of the genus Orthohantaviruses have an endemic distribution pattern [252,255]. Lesions associated with the nervous system, such as meningitis and encephalitis caused by hantavirus infection, are recorded much less frequently than other manifestations of the disease. However, there are data on cases of severe neurological manifestations associated with hantaviruses [256,257,258,259,260,261,262]. For example, in the work of Talamonti et al., a case of encephalitis due to hantavirus pulmonary syndrome caused by Andes virus infections is reported.

***Paramyxoviridae***. This family also includes regionally endemic chiropteran-associated viruses. Henipaviruses, which include **Hendra** (**HeV**) (Table 3, Figure 3a) and **Nipah** (**NiV**) (Table 3, Figure 3a) viruses, are relatively new paramyxoviruses of zoonotic origin that infect humans. HeV and NiV may be transmitted through bats, domestic pigs, horses, and from person to person [240,263]. Diseases associated with HeV and NiV viruses can affect a wide range of wild and domestic animals, as well as humans, in which these viruses exhibit a high level of pathogenicity, mainly causing pulmonary or encephalitic disease with an observed mortality of up to 60% and 90% for HeV and NiV, respectively [264]. In humans, the virus is transmitted through airborne droplets or direct contact with an infected person, infectious body fluids, or other secretions. Cases of infection have also been described after drinking date palm sap, because palm trees are a source of food for fruit mice of the family *Pteropodidae*, which are the natural hosts of the virus. The main areas of spread of the virus are India and Bangladesh. The consequences of viral infection include meningoencephalitis, the inflammation of the walls of blood vessels (systemic vasculitis), and severe respiratory failure [263].

***Poxviridae.* Monkeypox virus** (**MPV**) (Table 3, Figure 3b) has relatively few reported cases associated with nervous system complications of infection. Monkeypox is thought to rarely cause encephalitis, but Billioux et al. reported several cases of encephalitis in patients of different ages during outbreaks in Africa [265]. Reported clinical manifestations of infection during these outbreaks included encephalitis, ADEM, encephalomyelitis, and demyelinating encephalitis.

***Rhabdoviridae***. Bats (order *Chiroptera*) are the primary reservoir hosts for most lyssaviruses, while predators (order *Carnivora*) maintain rabies virus circulation; evolutionary analysis suggests that all lyssaviruses likely originated from bats [249,266]. Many viruses assigned to the genus are ubiquitous, but for some representatives of the genus, there is limited distribution and different areas of circulation [249,266]. Healthy bat populations may serve as reservoirs for the virus: for example, the prevalence of rabies virus (RABV) in large bat colonies is typically less than 1%, but 70% of bats can produce antiviral antibodies, suggesting that bats may be exposed frequently to allow them to produce immunity in the absence of infection [249].

The well-known neurotropic pathogen **rabies virus** (**RABV**) (Table 3) causes the majority of cases of rabies in humans worldwide and can be divided into two lineages: one transmitted primarily by carnivores with a worldwide distribution, and the other transmitted by bats and present only in America [133,249]. The disease caused by this pathogen is endemic in more than 150 countries and causes 40,000–70,000 deaths annually, most of which occur in Asia, Africa, and South America [240]. Human infection most often occurs through bites from sick dogs. Unfortunately, rabies is a disease with a high risk of death when severe clinical signs appear [263]. Cases of virus transmission during organ transplantation and perinatal transmission are also mentioned in the literature [263]. **Duvenhage virus** (**DUVV**), **Irkut virus** (**IRKV**), **Australian bat lyssavirus** (**ABLV**), **European bat lyssavirus 2** (**EBLV-2**), and **Mokola virus** (**MOKV**) also cause fatal diseases in humans, including those with central nervous system damage, including encephalitis and meningoencephalitis [267,268,269,270,271]. **European bat lyssavirus 2** (**EBLV-2**) (Table 3, Figure 3b) was discovered in bats in Europe; **Irkut virus** (**IRKV**) (Table 3, Figure 3b)—in Eurasia; **Duvenhage virus** (**DUVV**) (Table 3, Figure 3b)—in Africa; **Australian bat lyssavirus** (**ABLV**) (Table 3, Figure 3b)—in Australia [249]; and **Mokola virus** (**MOKV**) (Table 3, Figure 3b) is associated with shrews, rodents, and cats in Africa [267]. Unlike RABV, cases of infection with the viruses listed above are much less common; this is due to the predominance of competent viral hosts in certain endemic areas and, accordingly, the limitation of their spread.

**Table 3:** List of zoonotic viral pathogens causing neuroinflammation.

Family	Genus	Species	Common Names or Subspecies/ Acronym(s)	Genome	Host-Vector, Transmission	Geographic Distribution	NS Pathology	Reference
* Arenaviridae *	* Mammarenavirus *	* Mammarenavirus * *choriomeningitidis*	Lymphocytic choriomeningitis virus/ LCMV	ssRNA(+/−)	Hv: Predominantly wild and domestic rodents T: With bites; Contact with fomites/blood/nesting materials.	Worldwide (where rodents are present)	Meningitis, encephalitis, encephalomyelitis, meningoencephalitis, transverse myelitis	[46,49,62,81,247,272,273,274]
* Filoviridae *	* Orthoebolavirus *	* Orthoebolavirus * *zairense*	Ebola virus/ EBOV	ssRNA(−)	Hv: Non-human primates; bats; flying foxes; infected human hosts T: With bites; Contact with infectious body fluids of a patient (high risk group—medical workers); Contact with fomites/blood/nesting materials of infected animal hosts.	Imported infection: Mali, Nigeria, Senegal, Italy, Spain, UK, US, Russia (laboratory infection) Democratic Republic of Congo, Republic of the Congo, Gabon, Liberia, Sierra Leone, Guinea, South Africa	Meningitis, encephalitis, meningoencephalitis, neurocomplication after system infection	[248,274,275,276]
	* Orthomarburgvirus *	* Orthomarburgvirus * *marburgense*	Marburg virus/ MARV	ssRNA(−)	Hv: Egyptian fruit bat (*Rousettus aegyptiacus*), Sundevall’s leaf-nosed bat (*Hipposideros caffer*); non-human primates T: With bites; Contact with infected patients; Contact with fomites/blood/nesting materials of infected animal hosts.	Imported infection: Germany (Marburg, Frankfurt), Serbia (Belgrade), Russia (Koltsovo), Netherlands (Leiden), US, South Africa Angola, Democratic Republic of Congo, Kenya, Uganda, Zimbabwe, Guinea	Encephalitis	[112,250,277]
* Hantaviridae *	* Orthohantavirus *	* Orthohantavirus * *andesense*	Andes virus/ ANDV	ssRNA(−)	Hv: Wild and domestic rodents (long-tailed pygmy rice rat (*Oligoryzomys longicaudatus*)—most common host) T: With bites; Person-to-person transmission between humans (including breastfeeding, household contacts, nosocomial transmission); Contact with fomites/blood/nesting materials of infected animal hosts.	Highly endemic region: Regions of South America (Argentina, Bolivia, Chile, Uruguay)	Encephalitis	[257,278,279,280]
		* Orthohantavirus * *dobravaense*	Dobrava-Belgrade virus/ DOBV	ssRNA(−)	Hv: Rodents (yellow-necked mouse—*Apodemus flavicollis*; striped field mouse—*Apodemus agrarius*; Caucasian wood mouse—*Apodemus ponticus*; Small forest mouse—*Apodemus uralensis*) T: With bites and scratches; Inhalation of aerosolized droplets; Contact with fomites/blood/nesting materials of infected animal host; Contaminated food.	Russia (Central Russia, Western Siberia), Europe, Turkey Highly endemic region: Balkans	Encephalitis	[79,258,281,282]
		* Orthohantavirus * *puumalaense*	Puumala virus/ PUUV	ssRNA(−)	Hv: Rodents (bank vole—*Clethrionomys glareolus*) T: With bites and scratches; Inhalation of aerosolized droplets; Contact with fomites/blood/nesting materials of infected animal host; Contaminated food.	Russia (Central Russia, Western Siberia, Far East), Balkans, Europe (Northern, Western, Central regions)	Encephalitis, encephalomyelitis	[79,259,260,282,283]
		* Orthohantavirus * *seoulense*	Seoul virus/ SEOV	ssRNA(−)	Hv: Rodents (Norwegian brown rat—*Rattus norvegicus*; black rat—*Rattus rattus*) T: With bites and scratches; Inhalation of aerosolized droplets; Contact with fomites/blood/nesting materials of infected animal hosts; Contaminated food.	Far East of Russia, China, Japan, North and South Korea	Encephalitis	[79,262,282]
* Paramyxoviridae *	* Henipavirus *	* Henipavirus * *hendraense*	Hendra virus/ HeV	ssRNA(−)	Hv: Infected domestic animals (horses, pigs, dogs, cats); flying fox (fruit bats) (family *Pteropidinae*—*Pteropus alecto*, *Pteropus poliocephalus*, *Pteropus scapulatus*, *Pteropus conspicillatus*), etc. T: With bites and scratches; Contact with fomites/blood/nesting materials of infected animal hosts; Food-borne way (with horsemeat, pork, date palm sap or wine, fruits); Human-to-human.	Southeast Asia (including Singapore, Cambodia, Indonesia, Thailand, Malaysia, Philippines, Bangladesh), Eastern Australia, Ghana, Madagascar, Papua New Guinea, China, India, Latin America	Meningitis, encephalitis	[139,249,284,285,286,287,288]
		* Henipavirus * *nipahense*	Nipah virus/ NiV	ssRNA(−)	Hv: Infected domestic animals (horses, pigs, dogs, cats); flying fox (fruit bats) (*Pteropus giganteus*, *Pteropus hypomelanus*, *Pteropus lylei*); flying dog (*Cynopterus brachyotis*); cave nectar bat (*Eonycteris spelaea*); microbats (*Scotophilus kuhlii*, *Myotis*), etc. T: With bites and scratches; Contact with fomites/blood/nesting materials of infected animal hosts; Food-borne way (with horsemeat, pork, date palm sap, fruits); Human-to-human transmission.	Southeast Asia (including Singapore, Cambodia, Indonesia, Thailand, Malaysia, Philippines, Bangladesh), Eastern Australia, Ghana, Madagascar, Papua New Guinea, China, India, Latin America	Acute encephalitis	[112,249,284,285,286,288,289,290]
* Poxviridae *	* Orthopoxvirus *	* Monkeypox virus *	Monkeypox virus/ MPV	dsDNA	Hv: Non-human primates (mangabey monkeys), Gambian pouched rats, squirrels, prairie dogs T: With bites and scratches; Contact with fomites/blood of infected animal-host; Human-to-human transmission.	Highly endemic region: Tropical rainforest areas of Central and Western Africa Outbreaks (imported infection): 50 countries (worldwide)	Encephalitis, ADEM, encephalomyelitis, demyelinating encephalomyelitis	[139,263,265,291,292,293]
* Rhabdoviridae *	* Lyssavirus *	* Lyssavirus * *australis*	Australian bat lyssavirus/ ABLV	ssRNA(−)	Hv: Pteropod and insectivorous bat species (black flying fox—*Pteropus Alecto*; yellow-bellied sheath-tailed bat—*Saccolaimus flaviventris*) T: With bites and scratches; Contact with fomites (especially saliva)/blood of infected animal hosts.	Australia (New South Wales, the Northern Territory, Queensland, South Australia, Victoria, Western Australia)	Encephalitic rabies	[266,294,295]
		* Lyssavirus * *duvenhage*	Duvenhage virus/ DUVV	ssRNA(−)	Hv: Bats—presumably T: With bites and scratches; Contact with fomites (especially saliva)/blood of infected animal hosts.	South Africa; Europe (one case of imported infection—Netherlands)	Encephalitic rabies	[266,267,268]
		* Lyssavirus helsinki *	European bat lyssavirus 2/ EBLV-2	ssRNA(−)	Hv: Insectivorous microbats—Daubenton’s bat (*Myotis daubentonii*), pond bat (*Myotis dasycneme*) T: With bites and scratches; Contact with fomites (especially saliva)/blood of infected animal hosts.	Northeastern Europe, Mediterranean region, Netherlands, Switzerland, United Kingdom, Germany	Rabies-like encephalitis	[266,271,294,296]
		* Lyssavirus irkut *	Irkut virus/ IRKV	ssRNA(−)	Hv: Insectivorous microbats—greater tube-nosed bat (*Murina leucogaster*); domestic dog T: With bites and scratches; Contact with fomites (especially saliva)/blood of infected animal hosts.	Russia (Irkutsk region, Far East (Primorsky Krai region, Amur region)); China (Jilin province)	Encephalitic rabies	[294,297,298]
		* Lyssavirus mokola *	Mokola virus/ MOKV	ssRNA(−)	Hv: Domestic cats, dogs T: With bites and scratches; Contact with fomites (especially saliva)/blood of infected animal hosts.	Africa (Nigeria, Cameroon, Central African Republic, Ethiopia, Zimbabwe, South Africa)	Encephalitic rabies (human cases very rare)	[249,294,299]
		* Lyssavirus rabies *	Rabies virus/ RABV	ssRNA(−)	Hv: Vampire bat (*Desmodus rotundus*), big brown bat (*Eptesicus fuscus*), Mexican/Brazilian free-tail bat (*Tadarida brasiliensis*), silver-haired bat (*Lasionycteris noctivagens*), tri-colored bat (*Perimyotis subflavus*), carnivores (including domestic synanthrope species) T: With bites and scratches; Contact with fomites (especially saliva)/blood of infected animal hosts.	Worldwide	Meningoencephalitis, encephalitic rabies	[46,247,266,300,301,302,303]

Taxonomic and trivial names are given according to the reports of the International Committee on Taxonomy of Viruses (ICTV) https://ictv.global/msl and https://ictv.global/vmr (versions from 2022/2023); **ADEM**—Acute disseminated encephalomyelitis.

## 4. Widespread Viral Neuropathogens

In this section, we included ubiquitous viral pathogens that cause infections primarily not related to the nervous system (with the exception of polio enteroviruses and non-polio enteroviruses). A distinctive feature of these viruses, when compared with previously described pathogens, is their direct route of transmission from person to person, which usually does not include intermediate hosts.

***Adenoviridae***. Representatives of the family that cause various human diseases belong to the genus *Mastadenovirus* (Table 4) [304]. Mastadenoviruses are widespread and are detected in patients throughout the year, but the peak of incidence occurs in the winter–spring period [304,305,306]. All groups of the population are susceptible to infection, regardless of gender and age, but among immunocompetent individuals, young children are at greatest risk of infection and the development of severe complications [306,307,308].

The transmission of the virus occurs through contact with an infected person or through the inhalation of an aerosol containing the virus; by the fecal–oral route; or during the reactivation of a latent virus, which can persist in lymphoid and other tissues [304,306]. A rapid spread of the virus occurs in isolated populations through the close contact of virus carriers (including asymptomatic) and susceptible individuals in public places such as schools and health care facilities (nosocomial infection) [305,306].

About 100 variants of adenoviruses are known, with different tropism for tissues and circulating in different regions [304,309]. Adenoviruses typically cause mild infections of the upper or lower respiratory tract, gastrointestinal tract, or conjunctiva [305], but the most severe manifestations of infection, for example, pneumonia or meningitis, are observed in persons with immunosuppression, including drug-induced [304,305]. The spectrum of clinical manifestations of atypical severe infection varies and includes encephalitis, acute disseminated encephalomyelitis, cerebellitis, Guillain–Barré syndrome, acute flaccid myelitis, acute flaccid paralysis, and necrotizing encephalopathy, as well as symptomatic manifestations with status epilepticus [304,305,306,309,310,311].

***Astroviridae***. Astroviruses are widespread among various vertebrates, including humans. Human mamastroviruses (Table 4) are typical causative agents of acute intestinal infections, next in prevalence after rotaviruses and noroviruses (about 10% of cases), in children under 5 years old and immunocompromised patients [312,313,314,315]. However, recent studies have shown that a number of variants of astroviruses pathogenic for humans can also act as etiological agents of encephalitis and acute flaccid paralysis [313,314], including in immunocompetent persons [313]. Koukou et al. report a clinical case of encephalitis caused by the classic human astrovirus Virginia (HAstV) variant in a previously healthy 16-month-old girl. Also, in the work of Naccache et al., a case of fatal encephalitis in a child with X-linked agammaglobulinemia and a weakened immune system was described, caused by a variant of the human astrovirus Virginia, presumably of zoonotic origin [314].

***Coronaviridae***. Coronaviruses are found in many vertebrates. Coronaviruses pathogenic to humans (Table 4) (including zoonotic MERS and SARS) are endemic throughout the world and are currently classified into two genera, *Alphacoronavirus* and *Betacoronavirus*. The recent SARS-CoV-2 pandemic has significantly increased the interest of the medical and scientific community in the study of respiratory viruses and their potential to cause severe complications. Typically, in humans, coronavirus infection causes respiratory diseases: rhinitis, pharyngitis, bronchitis, and pneumonia [310,316]. However, severe manifestations of infection can also include serious complications in the nervous system. Human coronaviruses have been sensu lato recognized as neurotropic agents causing encephalitis, acute disseminated encephalomyelitis, Guillain–Barré syndrome, acute flaccid myelitis, necrotizing encephalopathy, etc. [310,316]. The mechanisms of penetration of coronaviruses and their interaction with nerve cells require further detailed study. However, already now, various studies and reviews speak about the role of these pathogens, in particular, using the example of SARS-CoV-2 [317,318,319,320].

***Flaviviridae***. The vast majority of *Flaviviridae* pathogens that cause diseases of the central nervous system are transmitted through the bites of certain insects and ticks; see Section 2 and Section 3. However, among the pathogens of this family that are transmitted from person to person without the mandatory participation of a carrier, one can note hepatitis C virus (HCV). Hepatitis C virus (HCV) (Table 4) is an important etiological agent of chronic hepatitis. Humans are the primary reservoir of the virus. In addition to its direct role in the development of hepatitis, HCV has also been identified as having a role in the development of other severe systemic concomitant diseases, including encephalitis, myelitis, and encephalomyelitis [38,321,322]. The mechanism of occurrence of CNS lesions during chronic HCV infection is not yet completely clear. But, for example, it has been suggested that during chronic infection, an autoimmune pathogenic mechanism occurs against blood vessels or myelin due to chronic viral antigenic stimulation, as has been observed in several patients with chronic HCV [321].

***Matonaviridae.*** The family includes only one genus, *Rubivirus*, to which Rubella virus (RuV) (Table 4), the causative agent of rubella, also known as “German measles”, belongs [323]. The transmission of the virus occurs through airborne droplets from person to person. Children are mainly susceptible to infection, and most cases are benign or asymptomatic [323,324]; however, this does not apply to congenital rubella, i.e., with maternal infection [324]. In postnatal infection, rubella virus may be the etiological agent of encephalitis in pediatric patients [81].

***Orthoherpesviridae***. Herpesviruses (HSV) (Table 4) are ubiquitous, occurring in many vertebrates [325]. In humans, herpesviruses are widely associated with diseases of the nervous system, such as aseptic meningitis, encephalitis, and meningoencephalitis [38,62,110,272,326,327]. After the primary infection, the adult host may develop a lifelong latent infection, and severe manifestations of the infection are observed, as a rule, in the fetus (with maternal infection) or in an organism with a weakened immune system [325]. For example, HSV encephalitis caused by HSV-1 occurs more often in immunocompetent adults, while HSV-2 affects immunocompromised individuals [328]. About 30% of cases of herpes encephalitis are associated with a primary infection (usually in children and teenagers), and 70% of cases are associated with the reactivation of the virus [110]. Also, some studies suggest and demonstrate data on the relationship of herpesviruses with the development of neurodegenerative diseases, such as Alzheimer’s disease and multiple sclerosis [110,327].

***Orthomyxoviridae***. The family includes ubiquitous viruses of various mammals and birds [329]. Well-characterized pathogens, including in humans, are Influenza A (IAV) (Table 4) and Influenza B viruses (IBV) (Table 4). In humans, influenza viruses mainly cause seasonal respiratory diseases, in some cases with serious complications. All groups of the population are susceptible to infection. It is believed that influenza viruses do not directly affect the central nervous system, but are neurotropic (for some variants) [330,331], and adverse pathological reactions are the result of the immune response to infection (secondary inflammation observed in the central nervous system) [38,332]. At the same time, the work of Popescu et al. describes cases of neurological complications in patients with influenza B virus, including myelitis, encephalitis, and Guillain–Barré syndrome [333]. The neuropathophysiology of influenza-associated neurological complications remains poorly understood but is thought to be related to systemic cytokine release and likely host-specific genetic predisposition [332]. Therefore, vigilance against influenza viruses is necessary in the development of neuropathologies, given the high variability and prevalence of these pathogens.

***Paramyxoviridae***. This family includes several genera of large heterogeneous enveloped RNA viruses that infect a variety of mammals, reptiles, birds, and fish [334]. Measles virus (measles virus) (Table 4) and human parainfluenza viruses (parainfluenza viruses) (Table 4) are causes of aseptic meningitis in extremely rare cases [335]. The mumps virus, before the widespread introduction of vaccines, was the most common cause of viral meningitis. However, whereas the incidence of mumps has gradually declined in developed countries, the virus continues to be a problem in developing regions [335]. Children are most susceptible to the disease [272]. Parainfluenza viruses, as well as the measles and mumps viruses, are transmitted through contact with a sick person through aerogenic means. The incidence of parainfluenza fluctuates throughout the year, with outbreaks recorded in the autumn and spring periods, in all age groups. In essence, the disease is characterized by damage to the upper respiratory tract; however, extrapulmonary manifestations of infection can manifest as meningitis and meningoencephalitis [336,337].

***Parvoviridae***. Members of the genera *Bocaparvovirus* (Table 4), *Erythroparvovirus* (Table 4), and *Tetraparvovirus* (Table 4) are associated with a wide range of conditions, including neurological complications [338]. *Human parvovirus B19* (B19V), a member of the genus *Erythroparvovirus*, is believed to cause aseptic viral meningitis relatively rarely [335]. In the paper of Vilmane et al., it was suggested that active parvovirus infection is associated with the development of meningitis and meningoencephalitis. It was shown that the genomic sequences of HBoV1–4 and B19V were present in 52.38% and 16.67% of samples from hospitalized patients with meningitis and meningoencephalitis of unknown etiology [339]. Also, the presence of obvious severe neurological symptoms, such as headache, disorientation, and impaired concentration, was more common in patients with confirmed parvovirus infection, which may likely indicate the connection of these viruses with the effect on the nervous system [339].

***Picornaviridae*** (Table 4) is a family of small icosahedral viruses containing positive-sense single-stranded RNA that cause subclinical infections in humans and animals or conditions ranging from mild fever to severe diseases of the heart, liver, and central nervous system [340]. Human-pathogenic members of the family included in this review, as etiological agents of neurological diseases, belong to the genera *Enterovirus*, *Hepatovirus*, and *Parechovirus*.

Representatives of the *Enterovirus* genus, are a well-studied ubiquitous pathogens, causing acute intestinal infections in children. Enteroviruses are the most common cause of viral meningitis in patients of any age [273]. In temperate regions, outbreaks, including reported cases of aseptic meningitis, tend to peak during the summer and autumn months, whereas in tropical regions, they occur consistently throughout the year [341]. Enteroviruses CV-B5, E-6, and E-30 are common causes of meningitis outbreaks worldwide [342], as is CVA2 [343]. Enteroviruses are the cause of most cases of viral encephalitis, acute flaccid myelitis [310], and acute flaccid paralysis [344], caused by non-polio variants of *Enterovirus C*—EV68 [310] and A71 [300], as well as CV-A1, CV-A11, CV-A13, CV-A17, CV-A19—A22, and CV-A24 [45]. It is also noted that infection in children of a young age can lead to delayed nervous development with motor, speech, and cognitive function impairment [45].

Representatives of the genera *Hepatovirus* (hepatitis A virus) and *Parechovirus* are also associated with the occurrence of viral meningitis [62,247]. Parechoviral meningoencephalitis is a problem among children under 5 years of age [345,346,347], with children under 6 months of age being at highest risk [347].

***Pneumoviridae*** (Table 4). This is a family of RNA-containing pleomorphic (mainly spherical or filamentous) enveloped viruses, including two representatives which are pathogenic for humans, *Human respiratory syncytial virus* (HRSV) and *Human metapneumovirus* (HMPV), classified in the genera *Orthopneumovirus* and *Metapneumovirus*, respectively [348]. HRSV, like HMPV, causes respiratory diseases in humans; however, in some cases, extrapulmonary complications can manifest as encephalitis, epilepsy, myelitis, and encephalomyelitis [310,349].

***Polyomaviruses***. Polyomaviruses (Table 4) are a family of small non-enveloped viruses, the genome of which is represented by double-stranded DNA, averaging 5 Mb in size, infecting various mammals and birds [350]. This family includes John Cunningham virus (JCV), of which more than half of the adult population are asymptomatic carriers (i.e., have a latent or persistent infection). During immunosuppression, viral reactivation can lead to the development of progressive multifocal leukoencephalopathy (PML), a disease characterized by multiple foci of demyelination of neurons in the brain. Now an early rare disease, it has become more common with the advent of acquired immunodeficiency syndrome (AIDS) [350], and also in combination with various immunosuppressive treatments [46].

***Retroviridae*** (Table 4). HIV infection leads to neurological complications in half of cases, including encephalitis [46]. In addition to encephalitis, HIV-associated neurocognitive disorder (HAND) is believed to remain a common cause of cognitive impairment, even in individuals treated with combination antiretroviral therapy (CART) [351]. HIV can enter the CNS early in infection, and persistent HIV infection and CNS inflammation are thought to contribute to the development of varying degrees of HAND [351], in approximately 50% of HIV-positive patients [352]. Latent HIV infection may persist in the brain even after systemic control is achieved using CART, thereby complicating and delaying treatment [351]. The pathogenesis of HAND involves either direct effects of the virus or the effects of viral proteins, and also the possible influence of antiretroviral drugs on amyloid metabolism, which in turn leads to changes in brain tissue [353].

**Table 4:** List of common viral pathogens that cause neuroinflammation.

Family	Genus	Species	Common Names or Subspecies/ Acronym(s)	Genome	Source, Predisposing Conditions, Transmission	NS Pathology	Reference
		(Sub)Species Complex/ Acronym(s)					
* Adenoviridae *	* Mastadenovirus *	* Human * *mastadenovirus A*	Human adenovirus 12/ HAdV-12	dsDNA	S: Infected human hosts Pc: Children under 5 y.o.; immunocompromised persons T: Inhalation of aerosolized droplets; Fecal–oral spread; Contact with infected tissue, water, environmental surfaces.	Meningitis, meningoencephalitis, encephalitis	[354]
		* Human * *mastadenovirus B*	Human adenovirus 3/ HAdV-3	dsDNA			[307,354]
			Human adenovirus 7/ HAdV-7	dsDNA			[307,354,355]
			Human adenovirus 11/ HAdV-11	dsDNA			[307,354]
			Human adenovirus 14/ HAdV-14	dsDNA			[307,354]
			Human adenovirus 16/ HAdV-16	dsDNA			[307,354]
			Human adenovirus 21/ HAdV-21	dsDNA			[307,354]
			Human adenovirus 34/ HAdV-34	dsDNA			[307,354]
			Human adenovirus 35/ HAdV-35	dsDNA			[307,354]
			Human adenovirus 50/ HAdV-50	dsDNA			[307,354]
			Human adenovirus 55/ HAdV-55	dsDNA			[354]
			Human adenovirus 66 ^1^;Human adenovirus B66 ^1^	dsDNA			[354]
			Human adenovirus 68 ^1^;Human adenovirus 3–16 ^1^	dsDNA			[354]
			Human adenovirus B79 ^1^	dsDNA			[354]
		* Human * *mastadenovirus C*	Human adenovirus 1/ HAdV-1	dsDNA	S: Infected human hosts Pc: Children under 5 y.o.; immunocompromised persons T: Inhalation of aerosolized droplets; Fecal–oral spread; Contact with infected tissue, water, environmental surfaces.	Meningitis, meningoencephalitis, encephalitis	[354]
			Human adenovirus 2/ HAdV-2	dsDNA			[354,355]
			Human adenovirus 5/ HAdV-5	dsDNA			[354,355]
			Human adenovirus 6/ HAdV-6	dsDNA			[354]
		* Human * *mastadenovirus D*	Human adenovirus 26; Adenovirus serotype 26/ HAdV-26	dsDNA	S: Infected human hosts Pc: Children under 5 y.o.; immunocompromised persons T: Inhalation of aerosolized droplets; Fecal–oral spread; Contact with infected tissue, water, environmental surfaces.	Meningitis, meningoencephalitis, encephalitis	[354]
			Human adenovirus 32/ HAdV-32	dsDNA			[354]
		* Human * *mastadenovirus E*	Human adenovirus 4/ HAdV-4	dsDNA			[354]
		* Human * *mastadenovirus F*	Human adenovirus 41/ HAdV-41	dsDNA			[308,354]
		unclassified *Human mastadenovirus* ^1^	Human adenovirus 76 ^1^	dsDNA			[354]
			Human adenovirus 77 ^1^	dsDNA			[354]
			Human adenovirus 78 ^1^	dsDNA			[354]
* Astroviridae *	* Mamastrovirus *	* Human astrovirus * ^1^	Human astrovirus Virginia| Pudget Sound ^1^/ HuAstV-PS ^1^	ssRNA(+)	S: Infected human hosts, HAI; zoonotic infection? Pc: Children under 5 years old (include immunocompetent), hereditary immunodeficiency, leukemia, HSCT, multiorgan dysfunction, immunocompromised patients T: Inhalation of aerosolized droplets; Fecal–oral spread; Contact with infected environmental surfaces.	Encephalitis	[356]
			Human astrovirus Virginia| Human-Mink-Ovine-like ^1^/ HAstV-VA ^1^|HMO-C-UK1(a) ^1^	ssRNA(+)		Encephalitis, progressive encephalitis, encephalopathy	[314,315,356]
			Human astrovirus Virginia| Human-Mink-Ovine-like ^1^/ HAstV-VA ^1^|HMO-C-PA ^1^	ssRNA(+)		Progressive encephalitis	[356]
			Human astrovirus Virginia| Human-Mink-Ovine-like ^1^/ HAstV-VA ^1^|HMO-C ^1^	ssRNA(+)		Encephalitis	[356]
			Human astrovirus Melbourne 2 ^1^/ HAstV-MLB2 ^1^; MLB2 ^1^	ssRNA(+)		Meningitis, acute meningitis	[356]
		* Mamastrovirus 1 *	Mamastrovirus 1/ MAstV1; HAstV ^1^	ssRNA(+)		Encephalitis, encephalopathy	[313]
		* Mamastrovirus 4 *	Mamastrovirus 4/ MAstV4; HAstV-4 ^1^	ssRNA(+)		Meningoencephalitis	[356]
* Coronaviridae *	* Alphacoronavirus *	* Human coronavirus 229E *	Human coronavirus 229E; Human coronavirus A ^1^/ HCoV_229E	ssRNA(+)	S: Infected human hosts Pc: Children under 5 y.o.; immunocompromised persons T: Inhalation of aerosolized droplets; Fecal–oral spread; Contact with infected tissue, water, environmental surfaces.	Encephalitis, ADEM	[310,316,335]
		* Human coronavirus NL63 *	Human coronavirus NL63; Human coronavirus A ^1^/ HCoV_NL63	ssRNA(+)			[310,335]
	* Betacoronavirus *	* Betacoronavirus 1 *	Human coronavirus OC43; Human coronavirus B ^1^/ HCoV_OC43	ssRNA(+)			[310,316,335]
		* Human coronavirus HKU1 *	Human coronavirus HKU1; Human coronavirus B ^1^/ HCoV_HKU1	ssRNA(+)			[310,335]
		* Middle East * *respiratory* *syndrome-related* *coronavirus*	Middle East respiratory syndrome-related coronavirus/ MERS-CoV	ssRNA(+)		Encephalitis, ADEM	[310,316,335]
		* Severe acute * *respiratory* *syndrome-related* *coronavirus*	Severe acute respiratory syndrome coronavirus	ssRNA(+)		Encephalitis, ADEM	[310,335]
			Severe acute respiratory syndrome coronavirus 2/ SARS-CoV-2	ssRNA(+)		Meningitis, encephalitis, ADEM, transverse myelitis, Guillain–Barré syndrome	[38,139,310,316,335,356]
* Flaviviridae *	* Hepacivirus *	* Hepacivirus * *hominis*	Hepatitis C virus/ HCV	ssRNA(+)	S: Infected human hosts Pc/T: Blood-borne transmission by drug-injection equipment, blood transfusion, organ transplantation; Genital contact; Congenital infection.	Peripheral neuropathy, disseminated encephalomyelitis, transverse myelitis, acute encephalitis	[38,321,322]
* Matonaviridae *	* Rubivirus *	* Rubivirus rubellae *	Rubella virus/ RuV	ssRNA(+)	S: Infected human hosts Pc/T: Children under 5 y.o.; Immunocompromised persons; Unvaccinated persons; Inhalation of aerosolized droplets; Congenital infection (TORCH).	Meningitis, acute encephalitis, progressive rubella panencephalitis	[52,81,357]
* Orthoherpesviridae *	* Cytomegalovirus *	* Cytomegalovirus * *humanbeta 5*	Human betaherpesvirus 5; Human cytomegalovirus/ HuBHV5, HCMV	dsDNA	S: Infected human hosts Pc/T: Children under 5 y.o.; Immunocompromised persons; Inhalation of aerosolized droplets; Congenital infection (TORCH).	Encephalitis, aseptic meningitis, neurodevelopmental deficits	[38,62,272,326,327]
* Orthoherpesviridae *	* Lymphocryptovirus *	* Lymphocryptovirus * *humangamma 4*	Human gammaherpesvirus 4; Epstein-Barr virus/ HuGHV4, EBV	dsDNA	S: Infected human hosts Pc/T: Inhalation of aerosolized droplets; Congenital infection.	Aseptic meningitis, meningitis, encephalitis, myelitis	[38,62,272,326,327]
	* Roseolovirus *	* Roseolovirus * *humanbeta 6a*	Human betaherpesvirus 6A; Human herpesvirus 6A/ HuBHV6A, HHV6A	dsDNA	S: Infected human hosts Pc/T: Inhalation of aerosolized droplets; Person-to-person transmission; Congenital infection.	Aseptic meningitis, encephalitis, multiple sclerosis	[38,62,326,327]
		* Roseolovirus * *humanbeta 6b*	Human betaherpesvirus 6B; Human herpesvirus 6B/ HuBHV6B, HHV6B	dsDNA		Aseptic meningitis, encephalitis, multiple sclerosis	[38,62,326,327]
		* Roseolovirus * *humanbeta 7*	Human betaherpesvirus 7; Human herpesvirus 7/ HuBHV7, HHV7	dsDNA		Aseptic meningitis, encephalitis, meningoencephalitis, vestibular neuritis	[62,247,327]
	* Simplexvirus *	* Simplexvirus * *humanalpha 1*	Human alphaherpesvirus 1; Herpes simplex virus type 1/ HuAHV1, HSV1	dsDNA	S: Infected human hosts Pc/T: Inhalation of aerosolized droplets; Person-to-person transmission; Congenital infection (TORCH).	Meningitis, meningoencephalitis, encephalitis, myelitis, Guillain–Barré syndrome	[38,110,327]
		* Simplexvirus * *humanalpha 2*	Human alphaherpesvirus 2; Herpes simplex virus type 2/ HuAHV2, HSV2	dsDNA			[38,110,327]
	* Varicellovirus *	* Varicellovirus * *humanalpha 3*	Human alphaherpesvirus 3; Varicella-zoster virus/ HuAHV3, VZV	dsDNA	S: Infected human hosts Pc/T: Person-to-person transmission; Congenital infection (TORCH).	Aseptic meningitis, encephalitis, meningoencephalitis, myelitis	[110,327,358,359,360]
* Orthomyxoviridae *	* Alphainfluenzavirus *	* Alphainfluenzavirus * *influenzae*	Influenza A virus/ IAV	ssRNA(−)	S: Infected human hosts Pc/T: Inhalation of aerosolized droplets	Meningitis, meningoencephalitis, encephalitis, myelitis, Guillain–Barré syndrome	[330,331,361]
	* Betainfluenzavirus *	* Betainfluenzavirus * *influenzae*	Influenza B virus/ IBV	ssRNA(−)			[333]
* Paramyxoviridae *	* Morbillivirus *	* Morbillivirus * *hominis*	Measles virus/ MV	ssRNA(−)	S: Infected human hosts Pc/T: Inhalation of aerosolized droplets	Encephalitis	[362,363,364]
	* Orthorubulavirus *	* Orthorubulavirus * *parotitidis*	Mumps virus; Mumps orthorubulavirus ^1^/ MuV	ssRNA(−)	S: Infected human hosts Pc/T: Inhalation of aerosolized droplets	Meningitis, encephalitis, myelitis	[365,366]
		* Orthorubulavirus * *laryngotracheitidis*	Human parainfluenza virus 2; Human orthorubulavirus 2 ^1^/ HPIV-2	ssRNA(−)	S: Infected human hosts Pc/T: Inhalation of aerosolized droplets	Encephalitis, severe acute encephalopathy	[367,368,369]
	* Respirovirus *	* Respirovirus * *laryngotracheitidis*	Human parainfluenza virus 1; Human respirovirus 1 ^1^/ HPIV-1	ssRNA(−)	S: Infected human hosts Pc/T: Inhalation of aerosolized droplets	Encephalitis, multiple sclerosis	[367,370,371]
		* Respirovirus * *pneumoniae*	Human parainfluenza virus 3/ HPIV-3	ssRNA(−)	S: Infected human hosts Pc/T: Inhalation of aerosolized droplets	Meningitis, encephalitis, Guillain–Barré syndrome	[367,369,372,373,374,375]
* Parvoviridae *	* Bocaparvovirus *	* Bocaparvovirus * *primate 1*	Human bocavirus 1/ HBoV1	ssDNA	S: Infected human hosts Pc/T: Inhalation of aerosolized droplets	Meningitis, meningoencephalitis, encephalitis	[339,376,377,378,379]
	* Erythroparvovirus *	* Erythroparvovirus * *primate 1*	Human parvovirus B19/ B19V	ssDNA	S: Infected human hosts Pc/T: Inhalation of aerosolized droplets	Meningitis, meningoencephalitis	[339,380,381,382]
	* Tetraparvovirus *	* Tetraparvovirus * *primate 1*	Human parvovirus 4/ PARV4	ssDNA	S: Infected human hosts Pc/T: Inhalation of aerosolized droplets	Encephalitis	[383]
* Picornaviridae *	* Enterovirus *	* Enterovirus A *	Coxsackievirus A2/ CVA2	ssRNA(+)	S: Infected human hosts Pc/T: Inhalation of aerosolized droplets; Fecal–oral spread; Contact with infected tissue, water, environmental surfaces.	Encephalitis, acute flaccid paralysis, aseptic meningitis	[45,384]
			Coxsackievirus A3/ CVA3	ssRNA(+)		Encephalitis, acute flaccid paralysis, aseptic meningitis	[45]
		* Enterovirus A *	Coxsackievirus A4/ CVA4	ssRNA(+)	S: Infected human hosts Pc/T: Inhalation of aerosolized droplets; Fecal–oral spread; Contact with infected tissue, water, environmental surfaces.	Encephalitis, acute flaccid paralysis, aseptic meningitis	[45]
			Coxsakievirus A5/ CVA5	ssRNA(+)		Encephalitis, acute flaccid paralysis, aseptic meningitis	[45,385]
			Coxsakievirus A6/ CVA6	ssRNA(+)		Meningoencephalitis, encephalitis, acute flaccid paralysis, aseptic meningitis	[45]
			Coxsakievirus A7/ CVA7	ssRNA(+)		Aseptic meningitis, encephalitis, acute flaccid paralysis	[386,387]
			Coxsakievirus A8/ CVA8	ssRNA(+)		Acute flaccid paralysis, aseptic meningitis	[45]
			Coxsakievirus A10/ CVA10	ssRNA(+)		Meningoencephalitis, encephalitis, acute flaccid paralysis, aseptic meningitis	[45]
			Coxsakievirus A12/ CVA12	ssRNA(+)		Acute flaccid paralysis, aseptic meningitis	[45]
			Coxsakievirus A14/ CVA14	ssRNA(+)		Acute flaccid paralysis, aseptic meningitis	[45]
			Coxsakievirus A16/ CVA16	ssRNA(+)		Encephalitis, meningoencephalitis, rhombencephalitis, acute flaccid paralysis, aseptic meningitis	[45,388]
			Enterovirus A71/ EV-A71	ssRNA(+)		Aseptic meningitis, acute flaccid myelitis/acute flaccid paralysis, encephalitis	[344,389,390,391,392,393,394]
		* Enterovirus B *	Coxsakievirus A9/ CVA9	ssRNA(+)	S: Infected human hosts Pc/T: Inhalation of aerosolized droplets; Fecal–oral spread; Contact with infected tissue, water, environmental surfaces.	Encephalitis, aseptic meningitis, meningoencephalitis, rhombencephalitis, acute flaccid paralysis, acute transverse myelitis	[45,389,395]
		* Enterovirus B *	Coxsackievirus B1/ CVB1	ssRNA(+)	S: Infected human hosts Pc/T: Inhalation of aerosolized droplets; Fecal–oral spread; Contact with infected tissue, water, environmental surfaces.	Encephalitis, aseptic meningitis, meningoencephalitis, rhombencephalitis, acute flaccid paralysis, acute transverse myelitis	[45,384]
			Coxsakievirus B2/ CVB2	ssRNA(+)		Encephalitis, aseptic meningitis, meningoencephalitis, rhombencephalitis, acute flaccid paralysis, acute transverse myelitis	[45,389,396]
			Coxsakievirus B3/ CVB3	ssRNA(+)		Encephalitis, aseptic meningitis, meningoencephalitis, acute flaccid paralysis, acute transverse myelitis	[45,389]
			Coxsakievirus B4/ CVB4	ssRNA(+)		Encephalitis, aseptic meningitis, meningoencephalitis, rhombencephalitis, acute flaccid paralysis, acute transverse myelitis	[45,389,397,398]
			Coxsakievirus B5/ CVB5	ssRNA(+)		Encephalitis, aseptic meningitis, meningoencephalitis, acute flaccid paralysis, acute transverse myelitis	[45,389]
			Coxsakievirus B6/ CVB6	ssRNA(+)		Aseptic meningitis, acute flaccid paralysis	[45]
			Echovirus 4/ E4	ssRNA(+)		Aseptic meningitis, encephalitis	[399,400,401,402]
			Echovirus 5/ E5	ssRNA(+)		Aseptic meningitis, encephalitis	[402,403]
			Echovirus 6/ E6	ssRNA(+)		Meningitis, encephalitis, Guillain–Barré syndrome	[389,402,404,405,406]
			Echovirus 7/ E7	ssRNA(+)		Meningitis, encephalitis, encephalomyelitis	[402,407,408]
			Echovirus 9/ E9	ssRNA(+)		Meningitis, encephalitis	[402]
			Echovirus 11/ E11	ssRNA(+)		Aseptic meningitis, encephalitis (HFMD)	[402,409,410]
			Echovirus 13/ E13	ssRNA(+)		Meningitis, encephalitis	[389,402,411]
		* Enterovirus B *	Echovirus 14/ E14	ssRNA(+)	S: Infected human hosts Pc/T: Inhalation of aerosolized droplets; Fecal–oral spread; Contact with infected tissue, water, environmental surfaces.	Aseptic meningitis, encephalitis	[402]
			Echovirus 15/ E15	ssRNA(+)		Aseptic meningitis, encephalitis	[389,402]
			Echovirus 16/ E16	ssRNA(+)		Aseptic meningitis	[402,412]
			Echovirus 17/ E17	ssRNA(+)		Aseptic meningitis, encephalitis	[402]
			Echovirus 18/ E18	ssRNA(+)		Aseptic meningitis, encephalitis	[402]
			Echovirus 19/ E19	ssRNA(+)		Aseptic meningitis, encephalitis	[389,402,413]
			Echovirus 22 ^1^	ssRNA(+)		Aseptic meningitis, Guillain–Barré syndrome	[402]
			Echovirus 25/ E25	ssRNA(+)		Aseptic meningitis, encephalitis	[402,414,415]
			Echovirus 30/ E30	ssRNA(+)		Meningitis, encephalitis	[364,380,381,402]
			Echovirus 31/ E31	ssRNA(+)		Aseptic meningitis	[402]
			Enterovirus B75/ EV-B75	ssRNA(+)		Aseptic meningitis, encephalitis	[416,417]
		* Enterovirus C *	Coxsackievirus A1/ CVA-1	ssRNA(+)	S: Infected human hosts Pc/T: Inhalation of aerosolized droplets; Fecal–oral spread; Contact with infected tissue, water, environmental surfaces.	Acute flaccid paralysis, aseptic meningitis	[45]
			Coxsackievirus A11/ CVA-11	ssRNA(+)		Acute flaccid paralysis, meningoencephalitis	[45]
			Coxsackievirus A13/ CVA-13	ssRNA(+)		Acute flaccid paralysis, aseptic meningitis	[45]
			Coxsackievirus A17/ CVA-17	ssRNA(+)		Acute flaccid paralysis, aseptic meningitis	[45]
			Coxsackievirus A19/ CVA-19	ssRNA(+)		Acute flaccid paralysis, aseptic meningitis	[45]
			Coxsackievirus A20/ CVA-20	ssRNA(+)		Acute flaccid paralysis	[45]
			Coxsackievirus A21/ CVA-21	ssRNA(+)		Acute flaccid paralysis, encephalitis, aseptic meningitis	[45]
			Coxsackievirus A22/ CVA-22	ssRNA(+)		Acute flaccid paralysis	[45]
			Coxsackievirus A24/ CVA-24	ssRNA(+)		Acute flaccid paralysis, aseptic meningitis	[45]
		Polioviruses ^1^ Include: Serotypes of the species *Enterovirus C* (types 1, 2 and 3 of wild *Poliovirus* (WPV))		ssRNA(+)	S: Infected human hosts Pc/T: Inhalation of aerosolized droplets; Fecal–oral spread; Contact with infected tissue, water, environmental surfaces.	Poliomyelitis, meningitis, aseptic meningitis	[418,419,420]
		* Enterovirus D *	Enterovirus D68; Human rhinovirus 87/ EV-D68	ssRNA(+)	S: Infected human hosts Pc/T: Inhalation of aerosolized droplets; Fecal–oral spread; Contact with infected tissue, water, environmental surfaces.	Meningo–myeloencephalitis, acute flaccid myelitis/acute flaccid paralysis	[20,344,421,422,423,424,425]
			Enterovirus D70/ EV-D70	ssRNA(+)		Acute flaccid myelitis	[392,426]
	* Hepatovirus *	* Hepatovirus A *	Hepatovirus A1; Hepatitis A virus/ HAV	ssRNA(+)	S: Infected human hosts Pc/T: Blood-borne transmission by drug-injection equipment, blood transfusion, organ transplantation; Genital contact; Congenital infection.	Encephalitis (extremely rare)	[427]
	* Parechovirus *	* Parechovirus A *	Human parechovirus 3/ HPeV-3	ssRNA(+)	S: Infected human hosts T: Inhalation of aerosolized droplets; Neonatal infection.	Meningitis, meningoencephalitis, encephalitis	[345,346,347,428]
* Pneumoviridae *	* Orthopneumovirus *	* Orthopneumovirus * *hominis*	Human orthopneumovirus; Human respiratory syncytial virus/ HRSV	ssRNA(−)	S: Infected human hosts T: Inhalation of aerosolized droplets	Meningitis, encephalitis (?), encephalopathy	[429,430,431,432,433]
	* Metapneumovirus *	* Metapneumovirus * *hominis*	Human metapneumovirus/ HMPV	ssRNA(−)	S: Infected human hosts T: Inhalation of aerosolized droplets	Encephalitis	[310,434,435,436,437,438]
* Polyomaviridae *	* Betapolyomavirus *	* Betapolyomaviru * *secuhominis*	JC polyomavirus; John Cunningham virus ^1^/ JC virus; JCV; JCPyV	dsDNA	S:/T: Autoinfection Pc: Immunocompromised condition	Meningitis, encephalopathy	[439,440]
* Retroviridae *	* Lentivirus *	* Human * *immunodeficiency* *virus 1*	Human immunodeficiency virus 1/ HIV-1	ssRNA-RT	S: Infected human hosts Pc/T: Blood-borne transmission by drug-injection equipment, blood transfusion, organ transplantation; Genital contact; Congenital infection.	Encephalitis (HAND)	[46,351]
		* Human * *immunodeficiency* *virus 2*	Human immunodeficiency virus 2/ HIV-2	ssRNA-RT	S: Infected human hosts Pc/T: Blood-borne transmission by drug-injection equipment, blood transfusion, organ transplantation; Genital contact; Congenital infection.	Encephalitis (HAND)	[441]

Taxonomic and trivial names are given according to the reports of the International Committee on Taxonomy of Viruses (ICTV) https://ictv.global/msl and https://ictv.global/vmr (versions from 2022/2023); ^1^ Commonly accepted alternative names not given in the reports of the International Committee on Taxonomy of Viruses (ICTV) (see above); **ADEM**—Acute disseminated encephalomyelitis; **HAI**—Hospital acquired infection; **HAND**—HIV-associated neurocognitive disorder; **HFMD**—Hand, foot, and mouth disease; **HSCT**—Hematopoietic stem-cell transplantation; **TORCH**—Acronym of hazardous for pregnancy infection agents: *Toxoplasma gondii* (or other agents like HIVs etc.), Rubella virus, Cytomegalovirus, Herpes simplex virus type 2.

## 5. Conclusions

In this review, we systematized and summarized information about human viral pathogens that can cause infections of the central nervous system. We based the systematization on the taxonomic affiliation of the corresponding infectious agents, dividing them into groups according to the predominant routes of transmission, accompanying the resulting list with information about their geographic distribution. In addition, special attention was paid to cases of infection with atypical pathogens, in order to present the most comprehensive list.

We hope that this work will be useful to healthcare professionals and researchers involved in this area and will contribute to the clarification of the etiological structure of viral neuroinfections.
